# Volatilomics-Based Microbiome Evaluation of Fermented Dairy by Prototypic Headspace-Gas Chromatography–High-Temperature Ion Mobility Spectrometry (HS-GC-HTIMS) and Non-Negative Matrix Factorization (NNMF)

**DOI:** 10.3390/metabo12040299

**Published:** 2022-03-28

**Authors:** Charlotte C. Capitain, Fatemeh Nejati, Martin Zischka, Markus Berzak, Stefan Junne, Peter Neubauer, Philipp Weller

**Affiliations:** 1 Institute for Instrumental Analytics and Bioanalytics, Mannheim University of Applied Sciences, 68163 Mannheim, Germany; c.capitain@hs-mannheim.de (C.C.C.); martin.zischka1@stud.hs-mannheim.de (M.Z.); markus.berzak1@stud.hs-mannheim.de (M.B.); 2 Bioprocess Engineering, Institute of Biotechnology, Technische Universität Berlin, Ackerstrasse 76 ACK 24, 13355 Berlin, Germany; f.nejati@tu-berlin.de (F.N.); stefan.junne@tu-berlin.de (S.J.); peter.neubauer@tu-berlin.de (P.N.)

**Keywords:** gas chromatography–ion mobility spectroscopy (GC-IMS), volatile organic compounds (VOCs), high-temperature ion mobility spectrometry (HTIMS), non-targeted screening (NTS) using machine learning, non-negative matrix factorization (NNMF), dairy fermentation, traditional and commercial kefir, quantitative polymerase chain reaction (qPCR)

## Abstract

Fermented foods, such as yogurt and kefir, contain a versatile spectrum of volatile organic compounds (VOCs), including ethanol, acetic acid, ethyl acetate, and diacetyl. To overcome the challenge of overlapping peaks regarding these key compounds, the drift tube temperature was raised in a prototypic high-temperature ion mobility spectrometer (HTIMS). This HS-GC-HTIMS was used for the volatilomic profiling of 33 traditional kefir, 13 commercial kefir, and 15 commercial yogurt samples. Pattern recognition techniques, including principal component analysis (PCA) and NNMF, in combination with non-targeted screening, revealed distinct differences between traditional and commercial kefir while showing strong similarities between commercial kefir and yogurt. Classification of fermented dairy samples into commercial yogurt, commercial kefir, traditional mild kefir, and traditional tangy kefir was also possible for both PCA- and NNMF-based models, obtaining cross-validation (CV) error rates of 0% for PCA-LDA, PCA-*k*NN (*k* = 5), and NNMF-*k*NN (*k* = 5) and 3.3% for PCA-SVM and NNMF-LDA. Through back projection of NNMF loadings, characteristic substances were identified, indicating a mild flavor composition of commercial samples, with high concentrations of buttery-flavored diacetyl. In contrast, traditional kefir showed a diverse VOC profile with high amounts of flavorful alcohols (including ethanol and methyl-1-butanol), esters (including ethyl acetate and 3-methylbutyl acetate), and aldehydes. For validation of the results and deeper understanding, qPCR sequencing was used to evaluate the microbial consortia, confirming the microbial associations between commercial kefir and commercial yogurt and reinforcing the differences between traditional and commercial kefir. The diverse flavor profile of traditional kefir primarily results from the yeast consortium, while commercial kefir and yogurt is primarily, but not exclusively, produced through bacterial fermentation. The flavor profile of fermented dairy products may be used to directly evaluate the microbial consortium using HS-GC-HTIMS analysis.

## 1. Introduction

Fermentation of food (such as milk) is an ancient practice for the preservation of food through the production of acids (such as lactic acid), alcohols, and possibly antimicrobial compounds [1]. Through the release of free amino acids, the synthesis of vitamins and the nutritional value of foods are enhanced by fermentation [2]. Furthermore, flavor compounds (such as acetaldehyde in yogurt and cheese) and other metabolites (such as extracellular polysaccharides), which will influence the organoleptic properties of the product, are formed. Traditionally, many fermented foods were produced by natural fermentation processes that involve the symbiotic fermentations of, for example, lactic acid bacteria and yeast [3]. Since each microorganism produces a versatile spectrum of flavor compounds, the composition of microorganisms influences the sensory properties, such as taste and flavor. For the development of new aroma profiles as well as the monitoring of cofermentations or the early detection of contaminants, holistic analysis tools are necessary, such as volatomic profiling by HS-GC-IMS. Being able to correlate certain substances with the presence of certain microorganisms, as shown in this paper by applying non-negative matrix factorization (NNMF) analysis, is a big step towards identifying microorganisms in food products purely based on their volatile organic compound (VOC) profile.

Due to the inherent diversity of biogenic samples, as observed in food analysis, and the chemical complexity of the sample matrices, analytical approaches covering a multitude of parameters, which are in parallel paired with strong discrimination power, are required. Analysis of the VOCs of samples, also known as VOC profiling, volatilomic profiling, or fingerprint analysis, allows for the detection of compounds in complex sample matrices without the need for detailed a priori knowledge of the molecular composition. Furthermore, VOC profiling can be completed without advanced sample preparation and without the need for detailed knowledge of the chemical constitution. GC-MS is commonly used for VOC analysis. However, due to its high sensitivity and resolving power on the one hand and its simplicity and robustness on the other, ion mobility spectrometry (IMS) has gained popularity for the analysis of VOCs [4]. In previous studies, the good stability and reproducibility of VOC profiling by HS-GC-IMS was demonstrated, resulting in relative standard deviations of retention time and drift time values of less than 1% [5]. Moreover, gas chromatography coupled with ion mobility spectroscopy (GC-IMS) has been shown to be an easy-to-handle and yet highly effective tool for VOC profiling [6].

The complexity of biological samples results from the presence of a variety of compounds, which, in their entirety, provide a characteristic GC-IMS spectrum, often referred to as the VOC profile or fingerprint [7,8]. Due to the large amount of data obtained by VOC profiling based on GC-IMS, machine learning tools are required for data analysis. In literature, these are often differentiated between targeted screening and non-targeted screening (NTS) approaches. Compared to the classical targeted approaches, where one or more chemical compounds are selected as markers, when applying NTS, the complete spectral data are analyzed using chemometric techniques. For this purpose, signals and signal intensities are the selected variables, without the prior identification of substances or the establishment of calibration curves. Since the identification of individual substances is relevant for model validation, the volatile compounds, which are responsible for class separation, are identified following the NTS analysis. As a result, non-targeted VOC profiling based on GC-IMS in combination with machine learning has emerged as a promising method for sample monitoring.

### 1.1. HS-GC-IMS for VOC Profiling

Since the 1970s, when IMS was first known as plasma chromatography, IMS has developed into a highly sensitive technique for the analysis of VOCs, which characterizes an ion’s mobility [9,10,11]. Due to its robust and easy-to-handle instrumentation, a wide range of application fields have been established for IMS today, such as food flavor analysis [4], process monitoring [12,13], and quality control [14], as well as the detection and quantification of warfare agents [15] and explosives [16,17].

With IMS, analytes are first ionized in the ionization region of the instrument [18]. Beta particles, which are emitted, for example, by a tritium (^3^H) source, initiate a gas phase reaction cascade of the drift gas (nitrogen or air), resulting in predominant proton–water clusters H^+^[H_2_O]_n_, which are commonly referred to as reactant ions [19]. The number of water molecules (n) depends on the gas temperature and the moisture content of the gas atmosphere [10]. Depending on the proton’s affinity, molecules entering the ionization region react with the reactant ions to produce protonated monomers MH^+^[H_2_O]_n−x_ while decreasing the intensity of the reactant ion peak (RIP). At higher analyte concentrations, proton-bound dimers M_2_H^+^[H_2_O]_m−x_ are generated by the attachment of additional analyte molecules. When the concentration further increases, the formation of higher molecular cluster ions, such as trimers or tetramers, is possible; however, due to their low stability and short lifetime, higher molecular cluster ions are rarely observed [20].

Following ionization, the analyte ions are transferred into the drift region through a gating mechanism based on a charged electrode. For precise control of the ion pulse width admitted into the drift tube, complex gating systems, such as Bradbury–Nielsen or field switching shutters, are employed [16]. In the drift tube, ions are accelerated towards the detector, a Faraday plate, and subsequently separated by their drift time (or mobility) in an electrical field at ambient pressure. The ions are slowed down by their collision with counter-flowing drift gas molecules in the collision cross section (CCS). The equilibrium between the acceleration generated by an electric field and the deceleration resulting from the collision with the drift gas molecules results in ions moving with a constant velocity towards the detector. Depending on their mass, charge, and spatial structure, the ions are separated in the drift tube and reach the detector at different drift times [21]. The drift time may be used to calculate the reduced ion mobility (K_0_) via the Mason–Schamp equation (see Equation (1)) [22].

(1) $$K_{0}=\frac{L}{E\times t_{D}}\times \frac{p}{p_{0}}\times \frac{T_{0}}{T}$$

where

K_0_ = reduced ion mobility in cm^2^ V^−1^ s^−1^
L = drift length in cm
E = electric field strength in V cm^−1^
t_D_ = drift time in s
p = pressure of the drift gas in hPa
p_0_ = ambient pressure: p_0_ = 1013.2 hPa
T = temperature of the drift gas in K
T_0_ = ambient temperature: T_0_ = 273.2 K

To avoid clustering in the ionization or drift region, IMS devices are commonly coupled to column separation techniques, such as liquid chromatography (LC) or gas chromatography (GC). Column separation coupled with drift time IMS separates analytes into two orthogonal ‘features’—first, the retention time through chromatography, and second, the drift time or mobility through IMS—resulting in a two-dimensional (2D) highly resolved GC-IMS spectrum [6,23]. In LC analysis, soluble compounds can be separated; however, sample preparation is a critical step for the data quality [24]. In GC analysis, the volatility of a sample is a prerequisite. Headspace (HS)-based techniques allow for the analysis of untreated samples, avoiding the time-consuming sample pre-treatment steps [5]. HS autosamplers are commonly used for GC sample injection. Being headspace-based, time-consuming sample pretreatment steps are usually not required for HS-GC-IMS analysis. HS-GC-IMS analysis can usually be carried out on untreated or almost untreated samples.

HS-GC-IMS has been demonstrated to be an effective technique for the evaluation of the VOC profiles of biological samples due to its simple system setup, robustness, and price [25,26,27,28,29]. Chemical profiling of food and beverages in combination with chemometric analysis is widely used for food authentication and ultimately to identify food adulteration and fraud [30]. Furthermore, the VOC profile is influenced by production processes as well as storage conditions. Consequently, process control and quality assurance, such as the control of food freshness and food safety, are topics of interest for NTS using HS-GC-IMS techniques [31,32]. While LC in combination with MS has been successfully applied for the investigation of specific biochemical and metabolic profiling [33,34], in this work, a GC setup was preferred due to its technical simplicity and simple sample preparation. In LC analysis, any soluble compound can be separated, but sample preparation is a critical step for the data quality [24]. Especially in combination with the ^3^H source of the IMS, the lack of solvents in GC analysis is advantageous to sensitivity and resolution.

### 1.2. Pattern Recognition and Dimension Reduction Techniques

Each fermented product displays a unique flavor profile, with a few compounds being characteristic of certain species of microorganisms or microbial consortia and other compounds being found in variable concentrations among many different dairy products. Since microorganisms secrete a variety of side metabolites, which often cannot be assigned to one specific microorganism, a comprehensive peak extraction method which enables the extraction of a wide range of compounds while minimizing potentially interfering co-extractives is needed. Since unspecific compounds are present [35], in this work, an NTS approach not requiring the specific identification of chemical marker sets prior to sample analysis was used. Due to the complexity of the flavor profile of microorganisms, a multivariate data analysis (MVA) approach was needed for sample analysis [36]. MVA approaches can be divided into exploratory, classification, and calibration methods. Exploratory methods, such as principal component analysis (PCA) or NNMF, are unsupervised and solely used for pattern recognition, whereas methods such as PCA-LDA, *k*NN, or PLS-DA are supervised methods used for classification.

PCA is a powerful technique for the unsupervised discovery of patterns in data which is further applied for dimension reduction [37]. The information extracted from a data matrix is explained through principal components (PCs), which are orthogonal (mathematically independent) to each other. Another dimension reduction technique which is less frequently used is NNMF. In NNMF analysis, a matrix X is factorized into two matrices W and H, with the requirement that all three matrices must contain only zero or positive elements. Therefore, the sample features must be positive values, such as those provided by HS-GC-IMS. Prior to factorization, a k-value, also known as a ‘rank of factors’, needs to be specified. The n-by-m matrix X is therefore divided into an n-by-k matrix W and a k-by-m matrix H. The factorization is not exact, as W × H is a lower-rank approximation of X. The factors W and H minimize the root mean square residual D between W and W × H. As NNMF decomposes samples into sums of their parts, NNMF models are, as opposed to PCA models, easily interpretable. Since PCA and NNMF models are developed without labels or prediction steps, they are generally considered unsupervised. Unsupervised statistical methods are exploratory methods that can be used to study data structures and search for clusters of samples [38]. Hierarchical cluster analysis (HCA) of PCA or NNMF models in a tree-like diagram (dendrogram) are, for instance, used to visualize multivariate association and sample similarities [39].

In comparison to unsupervised techniques, which provide predictions without labels or target variables, supervised techniques aim to build models able to predict target variables. In supervised learning, several data points or samples are described using predictor variables, or features, and target variables. For classification tasks, the scores obtained by unsupervised exploratory analysis are combined with subsequent supervised pattern recognition techniques to distinguish samples according to defined categories. Among the PCA-based qualitative methods are linear discriminant analysis (LDA) and *k*-nearest neighbors (*k*NN). Whereas PCA-LDA maximizes the separation of known categories, *k*NN assigns the category most common among the *k*-nearest neighbors. The disadvantage of using PCA-based methods is that the correlation between dependent and independent variables is not considered during PCA analysis, which can result in a loss of the information contained in higher PCs [37]. An alternative classification method is provided by partial least squares (PLS), where the scores are calculated by considering the relationship between the independent and dependent variables.

### 1.3. Microbial Composition and Flavor Profiles of Fermented Dairy

Yogurt is produced by bacterial fermentation of mixed cultures of *Lactobacillus delbrueckii* subsp. *bulgaricus* and *Streptococcus thermophilus*. Excreted products of the metabolism of these organisms are lactate, aroma compounds (including acetaldehyde and diacetyl), and eventually, exopolysaccharides [40]. In addition, other lactobacilli and bifidobacteria are sometimes added during or after yogurt production. Even after many years of commercially available yogurt, the aromatic profile produced by Lactobacillus delbrueckii subsp. bulgaricus and Streptococcus thermophilus is still the focus of current research. Here, volatiles are commonly identified and quantified by solid-phase microextraction and gas chromatography–mass spectrometry (SPME-GC-MS), often identifying more than 80 VOCs [41,42]. However, some disadvantages of SPME include a high coefficient of variation for certain setups, varying matrix characteristics, and interanalyte displacement effects caused by adsorption onto the fibers [43,44], which are unfavorable for NTS approaches.

Kefir is a fermented dairy product created through the symbiotic fermentation of milk by lactic acid bacteria and yeasts, with a slightly acidic taste and creamy consistency [3]. The composition of microorganisms differs strongly among kefir grains of different origin, resulting in a unique and versatile spectrum of flavor compounds which, in total, constitute the taste and flavor of kefir. Thus, different kefir varieties provide the opportunity to evaluate the milk fermentation by microbial consortia based on VOC profiling using HS-GC-IMS analysis.

Kefir originated in Caucasian countries and is considered one of the oldest fermented milk beverages [45]. Many beneficial health effects have been reported for kefir [46], including anti-inflammatory [47] and wound-healing properties [48]. Moreover, due to its association with probiotic bacteria [49] and its capacity to lower cholesterol levels [50], milk kefir has attracted increased attention of dairy producers and health-conscious consumers.

Traditionally, kefir is produced through natural fermentation by kefir grain, which is a conglomerate of microbial cells and their metabolites, coagulated milk proteins, and carbohydrates [51], such as the polysaccharide kefiran [52]. The exact microflora are not yet well defined and depend on the origin of the starter culture, conditions of growth (such as temperature, among others), processing of the milk, and type of milk used [53]. Furthermore, the microflora proportions change during the fermentation. While the kefir grains contain 83–90% lactic acid bacteria (including 53–65% of *Streptococci* and 24–33% of *Lactobacilli*) and 10–17% of yeast and acetic acid bacteria, the composition of the kefir was reported to be 92–96% of lactic acid bacteria (including 74–78% *Streptococci* and 15–20% *Lactobacilli*) and 4–8% of yeast [54].

The composition of microorganisms influences the sensory properties of kefir, which are dominated by lactic acid, volatile acids, diacetyl, carbon dioxide, and ethanol [55,56]. The metabolic pathway of lactic acid bacteria is distinguished between homolactic fermentation and heterolactic fermentation. Homolactic fermenters, such as *Streptococcus thermophilus*, *Lactobacillus lactis*, and *Lactobacillus bulgaricus*, mainly produce lactic acid from pyruvic acid, while heterolactic fermenters, such as *Leuconostoc* spp., produce ethanol in addition to lactic acid [57].

Furthermore, bacterial strains can produce alternative end products from pyruvic acid, such as formic acid and acetic acid or butane-2,3-diol. The latter is produced through the conversion of pyruvic acid to acetolactic acid and further to acetoin. While acetoin and butane-2,3-diol are more or less tasteless, their derivate diacetyl, which is produced by non-enzymatic chemical conversion from acetolactic acid, is an important flavor compound in dairy products [58]. The main catalytic by-product for energy production from carbohydates in yeast is ethanol [53,59]. For *Candida* spp., *Saccharomyces* spp., *Kluyveromyces* spp., and *Debaromyces* spp., flavor compounds such as ethanol, acetone, amyl-alcohol, and propanal have been reported [53]. Further sources for flavor and aroma compounds are lipid hydrolysis and protein hydrolysis. Lipolysis in milk results in the formation of free fatty acids, which are precursors to flavor compounds such as methyl ketones, alcohols, lactones, and esters. Methyl ketones, such 2-nonanone and 2-heptanone, which are attributed to blue cheese flavor, are, for example, formed by decarboxylation [60]. Yeast-mediated protein hydrolysis, for example, of casein results in the formation of small peptides and free amino acids which can be converted to alcohols, aldehydes, volatile acids, esters, and sulfur-containing compounds such as dimethyldisulfide and dimethyltrisulfide [61]. Aroma compounds such as isobutyrate, isovalerate, 3-methylbutanol, 2-methylbutanal, and 2-methylpropanal are formed from branched-chain amino acids [62].

For commercial dairy products, an extended shelf life and enhanced food safety are necessary. Therefore, the food industry has developed microbial starter cultures which produce bacteriocins for food preservation, enhance sensory properties (for example, through the production of organic acids and carbonyl compounds and the (partial) hydrolysis of proteins and fatty acids), and help to improve the functional and nutraceutical properties [63]. To ensure a longer shelf life of kefir, controlling metabolic activity and concomitant excessive production of carbon dioxide, which may cause expansion of the packaging container, is necessary. For easier handling and faster production, producers may prefer starter cultures which do not produce grains during the manufacturing process and which instead can directly be used as inoculum (semi-direct or direct-to-vat). The choice of yeast strains, however, contributes to the kefir’s flavor, and consequently, the characteristic properties of commercial products differ from traditionally made kefir [53].

### 1.4. Overall Research Objective

The objective of this work is the optimization of an headspace-gas chromatography–high-temperature ion mobility spectrometry (HS-GC-HTIMS) prototype for the differentiation of key compounds developed during the fermentation of dairy, including acetic acid, diacetyl, and ethyl acetate. Furthermore, two data reduction techniques (PCA and NNMF) are compared for their discrimination power of fermented dairy samples, based on VOC profiles obtained by optimized HS-GC-IMS. As NNMF is less commonly used for data processing and classification of VOC profiles, NNMF is evaluated as an alternative to PCA and PLS here. The performance of PCA and NNMF for kefir classification is determined in combination with machine learning tools (LDA, *k*NN, and support vector machines (SVM)), resulting in models with high prediction accuracy. Through backwards projection of NNMF-loadings, characteristic substances are identified, generating a flavor profile characteristic of each variety of fermented dairy. Lastly, the obtained flavor profile is correlated to its microbial composition to extract species-specific substances.

## 2. Results and Discussion

### 2.1. Optimization of VOC Profiling for Analysis of Fermented Foods

Preliminary analysis of fermented dairy samples using HS-GC-MS and HS-GC-IMS revealed some concerns related to overlapping peaks of ethanol, acetic acid, and ethyl acetate, as well as diacetyl. Since all four compounds are known to be key substances in dairy fermentation, an optimization of VOC profiling was conducted prior to NTS analysis.

The limitations of MS are mostly caused by the presence of ethanol. Ethanol is produced through natural fermentation and thus is naturally occurring in many matrices, such as fresh orange juice [64], honey [5], and olives [65]. In fermented foods, where the ethanol concentration may be even higher, the high abundance of ethanol can lead to a full saturation of the spectra during GC-MS analysis. To avoid the saturation of the spectra and to improve the sensitivity of the spectrometer, the scan range may be set to start above the molecular mass of ethanol. Therefore, the corresponding signals below a mass-to-charge (*m*/*z*) ratio of 47 are missing in the MS spectra [64]. Acetic acid (molecular mass 60.1 g mol^−1^), which is produced by acetic acid bacteria, such as acetobacter, has its main peaks at *m*/*z* ratios of 43 (100.0% relative intensity), 45 (90.4% rel. int.), and 60 (74.8% rel. int.). Furthermore, some peaks with a relative intensity below 20% can be found (see Supplementary Materials Figure S1A). Ethyl acetate (molecular mass 88.1 g mol^−1^), which is characteristic of yeast fermentation, has its main peak at a *m*/*z* ratio of 43 (100%) (see Supplementary Materials Figure S1B). Furthermore, three peaks with relative intensities between 13.7% and 14.9% at *m*/*z* ratios of 29, 45, and 61 are found. Further peaks show a relative intensity below 10%. The linear retention index (LRI) of acetic acid is 692, and for ethyl acetate, 609. Much closer LRIs were reported by Walsh et. al., 2016, obtaining an LRI value of 629 for acetic acid and 614 for ethyl acetate. As both compounds have very similar retention times, the similarity in *m*/*z* patterns makes identification nearly impossible when signals below a *m*/*z* ratio of 47 are missing.

An alternative to GC-MS analysis is provided by GC-IMS analysis. Compared to GC-MS data, which include *m*/*z* information, GC-IMS data are intrinsically limited to (normalized) drift times and retention times. In general, the advantage of IMS over MS is its simple and inexpensive design, primarily due to being operated at atmospheric pressure and hence not requiring vacuum pumps [10]. Furthermore, the use of radioactive ionization sources allows for portability, miniaturization, and mechanical robustness and therefore is suitable for field and benchtop applications [5]. Using reference substances, IMS spectra were obtained for ethanol, acetic acid, ethyl acetate, and diacetyl. Between one (for diacetyl) and four (for acetic acid) characteristic peaks were obtained per substance (see Supplementary Materials Figure S2A–D). The IMS drift time (center) and GC retention (start) time are listed in Supplementary Materials Table S1.

The first characteristic peak for ethanol (IMS drift time of 1.052 RIPrel) was more than 100 s long in the GC analysis, even at low concentrations. If the ethanol concentration is raised, the second peak at an IMS drift time of 1.141 RIPrel becomes dramatically enhanced as well (data not shown). This can cause a similar saturation issue as that discussed for GC-MS analysis. For acetic acid, a similar peak which showed a GC retention time of approx. 100 s was detected (peak 4). Furthermore, three distinct peaks were detected for acetic acid (peaks 1–3). The first and second characteristic peaks for acetic acid show a very close similarity to the characteristic peaks obtained for ethyl acetate. However, a third peak was found for acetic acid which can be used to differentiate between acetic acid and ethyl acetate. Unfortunately, this peak coincides exactly with the main peak of diacetyl. Therefore, it was concluded that neither GC-MS nor GC-IMS analysis would be able to differentiate alone between those three compounds. The detection of diacetyl using GC-MS was explicit (data not shown), and therefore, it was concluded that simultaneous GC-MS and GC-IMS analyses are needed for the discrimination of fermented dairy products. However, for the traditionally fermented kefirs, saturation at drift times of 1.052 RIPrel and 1.141 RIPrel is caused by an excessive abundance of ethanol (see Supplementary Materials Figure S2F). This saturation effect is dominant and interferes with the acetic acid peaks 3 and 4, as well as the diacetyl peak. A targeted analysis approach is likely to fail, and as such, a non-targeted screening approach is needed. However, it was still questionable whether a non-targeted screening approach alone would be able to distinguish between the four substances with the complication of ethanol saturation. In order to overcome the limitations set by the IMS method, an optimized IMS was needed.

**Figure 1:**
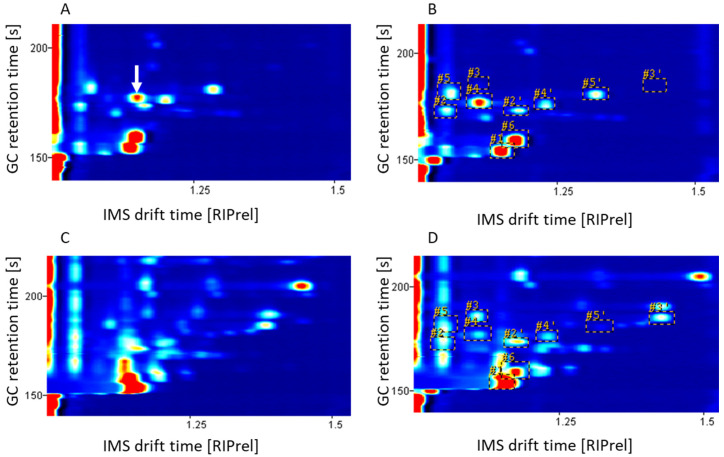
Temperature shift in prototypic high-temperature ion mobility spectrometer (HTIMS). (**A**) Commercial kefir at HTIMS cell temperature 90 °C, (**B**) commercial kefir at HTIMS cell temperature 120 °C, (**C**) traditional kefir LS (48 h) at HTIMS cell temperature 90 °C, and (**D**) traditional kefir LS (48 h) at IMS cell temperature 90 °C. Identified substances: #1, ethanol; #2, acetic acid; #3, ethyl acetate; #4, diacetyl; #5, 2-butanon; and #6, acetone. The arrow marks the diacetyl peak in close proximity to the acetic acid peak (#2′).

A prototypic GC-HTIMS, which was found to be more sensitive to acetic acid concentrations, was further used for kefir analysis. In Figure 1A,C, IMS spectra obtained at 90 °C using the prototypic GC-HTIMS are shown for commercial kefir from Mueller and traditionally fermented kefirs (LS, 48 h). The saturation effect due to the abundance of ethanol in traditionally fermented kefir, which was detected in the non-optimized GC-IMS analysis, is less dominant in the optimized GC-IMS spectra (see Figure 1C) but is still present for the earlier drift time of 1.052 RIPrel. To further reduce the interference of ethanol, removing ethanol prior to analysis is suggested.

The non-optimized GC-IMS analysis found four peaks between a GC retention time of 100 s and 125 s for the commercial kefir from Mueller (see Supplementary Materials Figure S2F), which were determined to be 2-butanone (two peaks) and diacetyl, as well as one unknown compound. The improved GC-IMS spectra show a retention time shift of approximately 65 s. Using the improved GC-IMS analysis, seven peaks were found in the corresponding area (GC retention time between 165 s and 185 s), which were attributed to 2-butanone (two peaks), diacetyl (one peak), a heterodimer (one peak), and acetic acid (two peaks), as well as one unknown compound (see Figure 1A). Thus, the improved method detected acetic acid, which was not detected previously. The acetic acid concentration of the commercial kefir was determined to be 276.52 mg L^−1^, and in the traditional kefir, 625.51 mg L^−1^. The IMS spectrum of the traditionally fermented kefir LS (see Supplementary Materials Figure S2F) shows three peaks which might be attributed to acetic acid. Since higher acetic acid concentrations were found for commercial kefir from Brandenburg (M1) and Andechser natur (M3), with 701.75 mg L^−1^ and 725.47 mg L^−1^ respectively, but no corresponding acetic acid peaks (data not shown), it was concluded that the peaks in the traditional kefir result from the presence of diacetyl and ethyl acetate instead. In conclusion, the sensitivity of non-optimized GC-IMS was not high enough to detect the acetic acid content in kefir.

**Figure 2:**
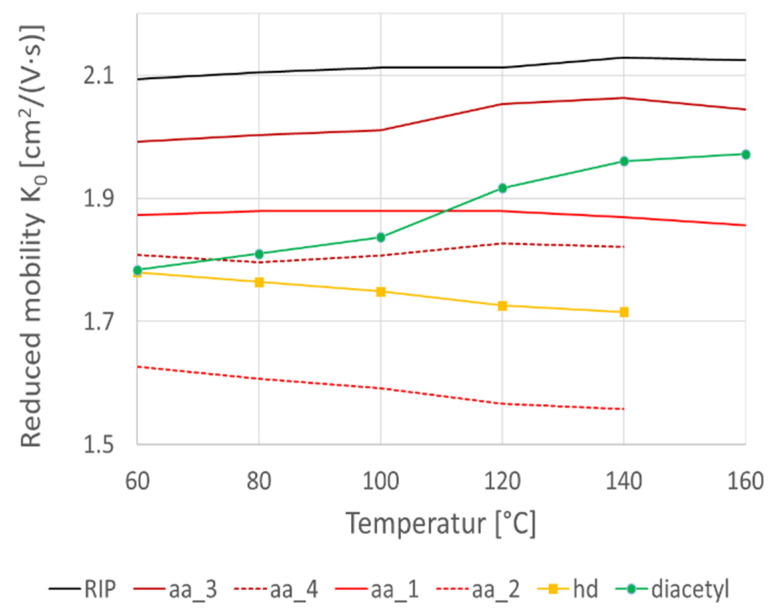
Reduced ion mobility (K_0_) of acetic acid (aa_1, aa_2, aa_3, and aa_4, red), diacetyl (green), and their heterodimer (hd, yellow) depending on the drift tube temperature (*x*-axis).

A prototypic GC-HTIMS was used to investigate the effect of elevated drift tube temperatures on the acetic acid and diacetyl resolution. A solution of 50 ppm acetic acid and 0.1 ppm diacetyl was investigated at drift tube temperatures of 60 °C, 80 °C, 100 °C, 120 °C, and 140 °C. K_0_ was calculated using the Mason–Schamp equation (see Equation (1)), with the results being shown in Figure 2. The RIP is almost temperature independent, with a nearly constant K_0_ value of 4 cm² V^−1^ s^−1^. For acetic acid, four peaks were detected (aa_1, aa_2, aa_3, and aa_4), which are displayed in red. Two peaks (aa_1 and aa_3) show a slight increase in K_0_, while the other two peaks (aa_2 and aa_4) seem to decrease with rising temperatures. For diacetyl, a single peak was detected (in green) which, in comparison, shows a steeply increasing K_0_ value with increasing temperature. Furthermore, a heterodimer (hd) was detected (displayed in yellow) which has a similar slope as the two acetic acid peaks (aa_2 and aa_4). At a drift tube temperature of 60 °C, the acetic acid peak aa_4, the heterodimer peak hd, and the diacetyl peak have nearly the same K_0_, whereby quantification is presumed to be difficult. With an increase in the drift tube temperature, these peaks drift apart, with maximum separation at 140 °C. At presumably 90 °C, the diacetyl peak coincides again with acetic acid peak aa_1. Therefore, the peaks are best separated at 120 °C and 140 °C. As the noise of the data increases with increasing drift tube temperatures (data not shown), 120 °C was found to be the optimal temperature for the simultaneous detection of acetic acid and diacetyl.

To further investigate the influence of the drift tube on the peak separation, commercial and traditionally produced kefir was analyzed at 90 °C and 120 °C (see Figure 1). At a drift tube temperature of 90 °C, the diacetyl peak and the heterodimer peak (acetic acid and diacetyl) are in close proximity to the acetic acid peak (see Figure 1A, white arrow). To address this problem, the drift tube temperature was raised to 120 °C. After increasing the temperature to 120 °C (see Figure 1B), the diacetyl peak moves towards lower drift times, away from the acetic acid peak. This temperature shift was also applied to the traditionally fermented kefir. Due to the ethanol saturation, the diacetyl and acetic acid peaks overlap at a drift tube temperature of 90 °C (Figure 1C). After increasing the temperature to 120 °C (see Figure 1C), the diacetyl peak moves towards lower drift times, away from the acetic acid peak. The temperature shift therefore decreases overlapping and makes the detection of diacetyl and acetic acid possible. The IMS drift time (center) and GC retention (start) for characteristic peaks found in the optimized HS-GC-IMS analysis at an IMS cell temperature of 120 °C are listed in Supplementary Materials Table S2.

### 2.2. Kefir Discrimination by PCA and NNMF

To investigate the microbial consortium of commercial yogurt and kefir, as well as traditionally made kefir, 76 kefir and yogurt samples were analyzed in this study using an optimized HS-GC-HTIMS prototype. The traditional kefir derived from two mild varietes (FN and LS) and three tangy varietes (PN1–3). The dataset was divided into training and test data, using a train–test split of 80 to 20. To study the data structure profiles, exploratory methods, including unsupervised PCA and NNMF, were applied for the data processing of VOC profiles obtained from 61 fermented dairy samples. HCA was used to visualize sample similarities.

A mean-centered PCA was carried out to identify chemical differences among the 3D HS-GC-IMS data matrices (61 samples × 612 chromatographic retention time values × 531 ion mobility drift time values) to detect possible clusters within sample sets. The first principal component (PC) explains 69.5%, the second PC 12.5%, the third PC 3.8%, and the fourth PC 3.3% of the total variance in the original information. As projections of any two PCs obtained from multidimensional data onto a 2D space may lead to overlapping clusters in one plot, a 3D plot was chosen for visualization. The data structure in a reduced dimension determined by PCA is visualized in Supplementary Materials Figure S3, showing a 3D scatter plot of PC1, PC2, and PC4, explaining 85.3% of the total variance. This projection determines four groups (commercial yogurt, commercial kefir, traditional mild and traditional tangy kefir) which are well-separated and grouped together with only a slight overlap between samples. PC1 differentiates between commercial and traditional samples, PC2 between traditional kefirs (mild and tangy), and PC4 between commercial samples (yogurt and kefir). The results of unsupervised PCA analysis demonstrate that the fermented dairy samples for all the categories have different characteristics and feature different VOC profiles in a multidimensional space. Thus, the HS-GC-IMS spectra contain valuable information for discriminating between products based on VOC profiling.

**Figure 3:**
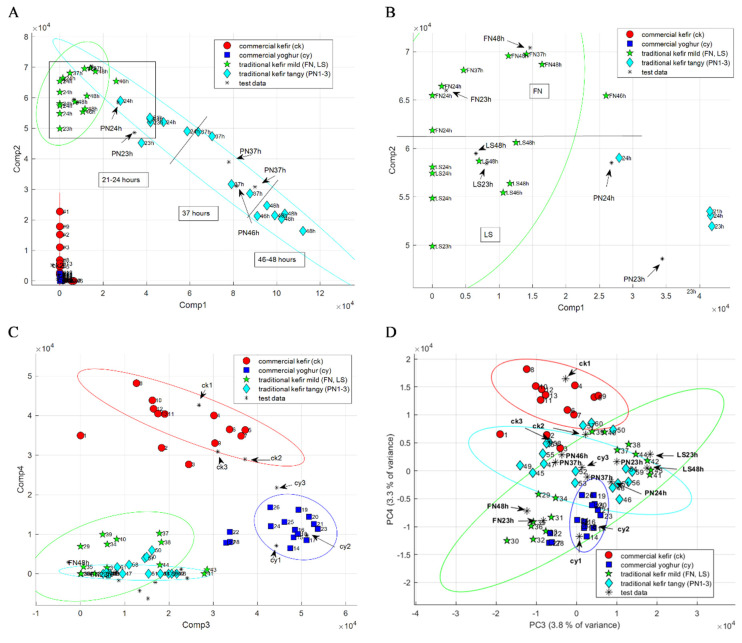
Scatter plot based on non-negative matrix factorization (NNMF) and principal component analysis (PCA) of preprocessed HS-GC-HTIMS spectra of fermented dairy, showing (**A**) NNMF-C1 and NNMF-C2, (**B**) NNMF-C1 and NNMF-C2 (enlarged), (**C**) NNMF-C3 and NNMF-C4, and (**D**) PC3 and PC4.

An alternative method to PCA for pattern recognition and dimensional data reduction is provided by NNMF, which similarly decomposes samples into sums of their parts. NNMF was carried out to identify clusters of chemical markers that differ among the 3D HS-GC-IMS data matrix. The k-value, which needs to be set prior to analysis, determines the number of main components into which the data matrix will be divided. To ensure comparability with PCA analysis, the k-value for NNMF analysis was set to 4. Figure 3A–C shows 2D scatter plots for the first four components (Cs) determined by NNMF, through which a visualization of the data structure in a reduced dimension is obtained. Figure 3A shows a 2D scatter plot of NNMF-C1 and NNMF-C2, obtained from NNMF analysis of preprocessed HS-GC-IMS spectra of fermented dairy. Figure 3C shows a 2D scatter plot of NNMF-C3 and NNMF-C4. While PCA shifts the data samples so that they have a mean of 0, NNMF expresses images as combinations of patterns. Therefore, the resulting components do not show differences between groups, but characteristics of a single group. Like in PCA analysis, NNMF projection determined four groups (commercial yogurt, commercial kefir, and traditional mild and traditional tangy kefir) which are well-separated and grouped together with only a slight overlap between samples. NNMF-C1 shows characteristic patterns of traditional tangy kefir, NNMF-C2 of traditional mild kefir (see Figure 3A), NNMF-C3 of commercial yogurt, and NNMF-C4 of commercial kefir (see Figure 3C). Furthermore, a clear separation of the two different types of traditional mild kefir (LS and FN) are observed (Figure 3B), as well as a time-wise grouping for the tangy kefir (21–24 h, 37 h, and 46–48 h). A closer look at PC3 and PC4, which is shown as a 2D scatter plot in Figure 3D, obtained a similar separation between the determined subgroups. PC3 shows a time-wise grouping for the tangy kefir (21–24 h, 37 h, and 46–48 h), and PC4 differentiates between the two different types of traditional mild kefir (LS and FN).

Unsupervised statistical methods are exploratory methods that can be used to study data structures and identify sample clusters [38]. To visualize the clusters of fermented dairy samples, HCA was used for the PCA and NNMF models. In HCA, a tree-like diagram (dendrogram) visualizes the multivariate sample similarities. The vertical distance on the dendrogram equates to similarities between merging clusters.

**Figure 4:**
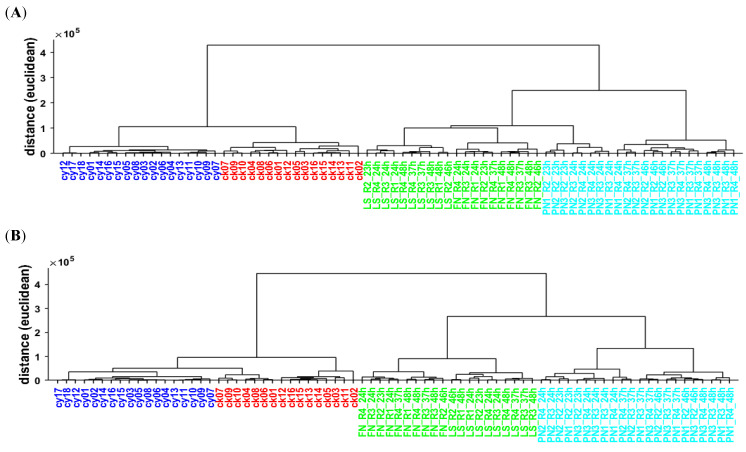
Dendrogram based on (**A**) PCA analysis (PC1–PC4) and (**B**) NNMF analysis (C1–C4). A detailed description of the sample data is provided in Appendix A, Table A1, Table A2 and Table A3.

The results of the HCA analysis performed on PCA and NNMF are shown in Figure 4. A dendrogram based on PCA analysis and NNMF analysis is shown in Figure 4A,B, respectively. At a glance, both dendrograms appear similar, and the four main groups appear salient. For both PCA and NNMF, commercial yogurt and commercial kefir show short distances within their clusters, which suggests little sample variation. Furthermore, both dendrograms display the subgroups of LS, FN, PN earlier harvest, and PN later harvest. However, there are also some differences between PCA- and NNMF-based analysis. The PCA-based analysis shows a stronger affiliation of PN early harvest with the traditional mild kefirs (LS and FN), compared to the more aged PN kefir, while NNMF analysis separates LS and FN from all PN samples in the first cluster. The comparison between PCA and NNMF analysis shows that both techniques are able to detect characteristics and features within the multidimensional spectral space. Both methods result in a clear separation between the four main groups and are able to detect meaningful subgroups based on the VOC profiles obtained by HS-GC-IMS analysis.

### 2.3. Comparison of PCA and NNMF for Kefir Classification Based on HS-GC-IMS Data

After successful discrimination of HS-GC-IMS data using PCA and NNMF, the resulting models were applied for kefir classification. For classification, LDA, *k*NN, and SVM were used in combination with PCA and NNMF. PCA-based models are prone to overfitting, which occurs when residual noise is included in the model fitting and class separation is predicted falsely. To minimize overfitting, the number of PCs should be adjusted carefully to the data. The ideal number of PCs used for each PCA based model was determined based on the lowest classification error rate by cross-validation (CV), calculated for 2 to 10 PCs. As shown in Table 1, for the prediction of four classes (commercial yogurt, commercial kefir, traditional mild kefir, and tangy kefir), robust models were built using the first four PCs. It should be noted that while an almost perfect separation between classes was obtained using PC1, PC2, and PC4 (see Supplementary Materials Figure S3), the third PC significantly improved the classification accuracy and was therefore included here (data not shown).

The CV error rates for PCA-LDA and PCA-*k*NN (*k* = 5) using four PCs to predict four labels were 0%, and for PCA-SVM, 3.3%. To further validate the PCA-based models, a validation set with 15 external measurements was used (3 commercial yogurt, 3 commercial kefir, 4 traditional mild kefir, and 5 traditional tangy kefir). For all three models, a perfect prediction accuracy of 100% was obtained. The CV error rates for NNMF-LDA, NNMF-*k*NN (*k* = 5), and NNMF-SVM using four components to predict four labels were 3.3%, 0%, and 5%, respectively. The predictive accuracy obtained for the NNMF-based models were 87% for NNMF-LDA and 100% for NNMF-*k*NN (*k* = 5) and NNMF-SVM. Using *k*NN, both models (PCA- and NNMF-based) showed perfect CV scores and prediction accuracy, as well as higher CV errors using SVM. While LDA showed perfect results in combination with PCA, both the CV error rate and the prediction accuracy were significantly worse in combination with NNMF. As an additional method, PLS discriminant analysis (PLS-DA) was evaluated for the use of kefir classification. Using the first four PLS components, the CV error rate was 1.6%. Using five PLS components, the CV error rate was reduced to 0%. Both models showed a perfect predictive accuracy of 100% when tested on the unseen validation measurements.

The discriminant analysis of kefir using both PCA and NNMF suggests that the mild kefirs FN and LS can be separated into two individual groups. Therefore, PCA and NNMF analysis were subsequently used to predict five labels using four and five components. In PCA analysis, the different PCs are sorted by the variance that they explain. Therefore, early PCs have a high impact on the result, and later PCs may have a small impact. Therefore, only minor changes are observed in a dendrogram based on PCA analysis with five groups and five labels compared to a dendrogram based on PCA analysis with four groups and four labels (data not shown). The components obtained by NNMF analysis, on the contrary, are not sorted by importance. As a result, the number of groups used for NNMF analysis may have a significant impact on the discrimination results. For visualization purposes, in Supplementary Materials Figure S5, a dendrogram based on NNMF analysis with five groups and five labels is shown. When comparing this dendrogram to a dendrogram based on NNMF analysis with four groups and four labels (see Figure 4B), major changes in allocation were observed. The PN samples from the earlier harvest, for example, which were previously part of the PN branch, are now allocated next to the LS kefir.

The CV error rate and prediction accuracy for the prediction of five labels are shown in Table 2. As only ten measurements were performed for each type, out of which eight were part of the training set and two were of the validation set, an unbalanced number of measurements resulted between groups. As a result, only 8-fold CV was possible. In general, small sample sizes and unbalanced classes can cause problems in statistical analysis. Small sample sizes are often associated with higher reported classification accuracy, and unequal sample sizes may result in a type 1 error (false positive) [66]. In particular, k-fold CV can produce strongly biased performance estimates with small sample size and machine learning [67]. Better suited for small sample sizes is, for example, external validation using a train–test split approach, as it generates robust and unbiased performance estimates [67]. Following this, both k-fold CV and external validation were applied. For PCA-*k*NN using four PCs, a perfect score was obtained for five labels, while a slightly higher CV error was found for PCA-LDA. Using five PCs, however, the CV error rate for PCA-LDA decreased to 0%. Both PCA-SMV-based models showed significantly worse CV error rates, and for PCA-SVM with five PCs, the prediction accuracy dropped to 93%. For NNMF-based models using five components to predict five labels, both LDA and SVM obtained comparably poor CV error rates and prediction accuracies, while *k*NN maintained a perfect score.

For PLS-DA, a perfect score was obtained for both four and five components. The comparison of PCA-based models and NNMF-based models shows that PCA has a slight advantage for the classification of kefir based on HS-GC-IMS data. While NNMF only obtained perfect scores in combination with *k*NN, for PCA based models, both LDA and *k*NN obtained perfect scores for the prediction of four and five labels. In conclusion, our results demonstrate that PCA, NNMF, and PLS are well-suited multivariate statistic tools for the discrimination, classification, and prediction of fermented dairy products based on VOC profiling by HS-GC-IMS. No issues regarding small sample sizes or unbalanced classes were detected.

### 2.4. Backward Projection of Loadings Using Four NNMF Components and Substance Identification

PCA analysis shifts the data to obtain a mean of 0; thereby, differences between groups are expressed by the different components. Through the back-projection of loadings, these differences can be visualized as HS-GC-IMS spectra. In contrast to PC1 and PC2, which differentiate between single groups (PC1 differentiates between commercial and traditional samples and PC2 between traditional kefirs), PC3 and PC4 differentiate between several groups. As shown in Figure 3D, PC3 shows a time-wise grouping for the tangy kefir (21–24 h, 37 h, and 46–48 h) as well as a differentiation between the two different types of traditional mild kefir (LS and FN). Furthermore, PC4 also differentiates between the two different types of traditional mild kefir (LS and FN) but at the same time differentiates between commercial yogurt and commercial kefir. A loadings plot obtained for PC4, for example, shows differences between commercial yogurt and commercial kefir as well as differences between the two types of traditional mild kefir (LS and FN). Consequently, the substances detected through PC3 or PC4 are not easily interpretable.

NNMF expresses spectra as combinations of patterns, and the resulting components do not show differences between groups, but rather characteristics of a single group or a combination of groups. The order of the component does not allow for drawing conclusions about the importance of each group. The composition of groups is dependent on the number of components chosen and the initial seed for H0 and W0. As discussed in Section 2.2, NNMF-C1 shows characteristic patterns of traditional tangy kefir, NNMF-C2 of traditional mild kefir, NNMF-C3 of commercial yogurt, and NNMF-C4 of commercial kefir (see Figure 3A–C). Semi-quantitative results are presented as a heatmap in Supplementary Materials Figure S4. A detailed list of substances detected in this work is provided in Table 3.

**Figure 5:**
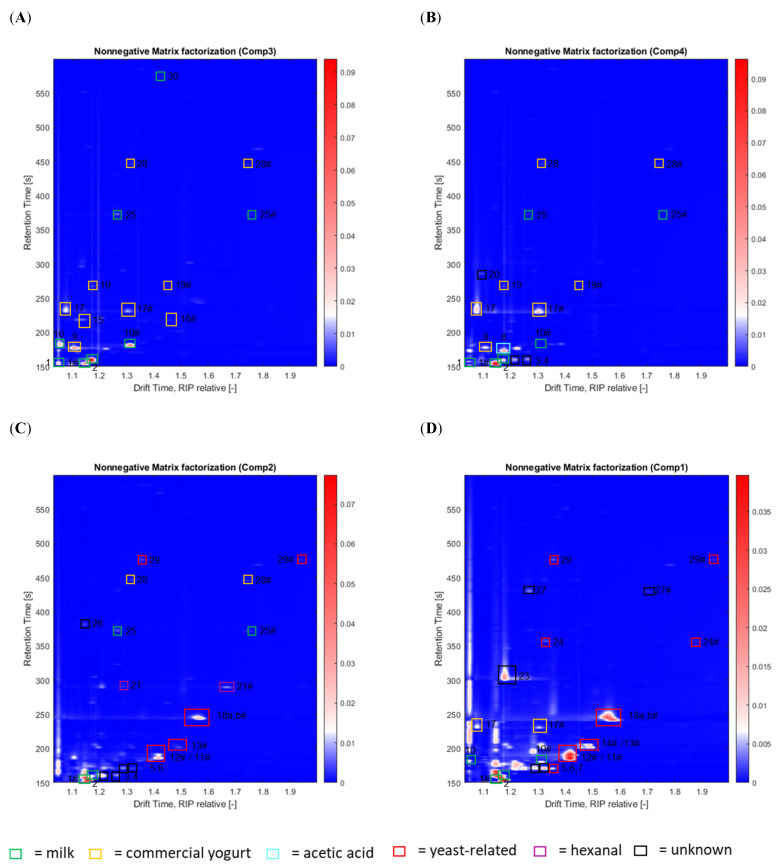
Loadings plot for NNMF analysis with *k* = 4, showing (**A**) component 3, (**B**) component 4, (**C**) component 2, and (**D**) component 1. Milk-related compounds are marked in green, commercial yogurt in orange, acetic acid in turquoise, yeast-related compounds in red, hexanal in purple, and unknown compounds in black.

Through the back-projection of loadings, the characteristic components of groups 1 to 4 are visualized as HS-GC-IMS spectra (see Figure 5A–D). The substances were identified using GC-MS and GC-IMS. Using GC-MS, eight substances were identified, including acetone, 2-methyl propanal, acetic acid, butane-2,3-dione (diacetyl), ethyl acetate, 2-methyl-1-propanol, 3-methylbutanal, and 3-methyl-1-butanol. Furthermore, 16 substances were identified using GC-IMS in combination with reference substances. Several other compounds, including some substances that are commonly found in fermented dairy, were tested for with reference substances, but not found in this work: Six alcohols (butanol, pentanol, hexanol, heptanol, octanol, and 2,3-butanediol) were not detected, and neither were five alkanes (hexane, heptane, octane, nonene, and decane), seven aldehydes (propanal, butanal, pentanal, heptanal, octanal, nonanal, and benzyaldehyde), two ketones (2-hexanone and 2-octanone), four esters (ethyl propanoate (previously reported by Wang and coworkers [74]) and ethyl 2-methylpropanoate (previously reported by Wang and coworkers [74]), ethyl butyrate (previously reported by Walsh and coworkers [68]), and butyl acetate), two acids (propanoic acid and pentanoic acid), or two sulfuric compounds (dimethyl disulfide and dimethyl trisulfide).

NNMF-C3 (see Figure 5A) includes five compounds which were also found in milk (data not shown): ethanol (1), acetone (2), 2-butanone (10), 2-heptanone (25), and 2-nonanone (30). Since all five compounds were already found in milk in the same concentrations, they did not develop through fermentation. Six additional compounds were found in NNMF-C3, including two diketons (butane-2,3-dione (9) and pentane-2,3-dione (15)) and two ketones (2-pentone (16) and acetoin (17)), as well as two acids (butyric acid (19), hexanoic acid (28)). Therefore, all six compounds are characteristic of yogurt, with the highest amounts being butane-2,3-dione (diacetyl) and acetoin. Diacetyl is produced by non-enzymatic chemical conversion from acetolactic acid, which has been reported as an important flavor compound in dairy products [58]. It is naturally produced by lactic acid bacteria [75]. Furthermore, the formation of diacetyl and its tasteless derivate acetoin derives from yeast [59]. As many bacteria cannot convert acetoin to diacetyl, acetoin is a typical by-product from bacterial fermentation. As NNMF-C3 is characteristic of yogurt, it was concluded that the obtained loadings are characteristic of yogurt.

NNMF-C4 (see Figure 5B), which is characteristic of the commercial kefir group, shows strong similarities to NNMF-C3. The concentration of 2-butanone (10) was decreased compared to milk, which is an indication of assimilation by microbes. Furthermore, the concentration of acetoin (17) had elevated 3.5-fold, while pentane-2,3-dione (15) and 2-pentone (16) were missing. The abundance of butane-2,3-dione (9) had decreased 2.3-fold. Butane-2,3-dione and pentane-2,3-dione are considered characteristic yogurt flavors [76], and therefore, their reduction and absence, respectively, are an important finding for the differentiation between commercial kefir and yogurt. Acetoin has a similar flavor to butane-2,3-dione, with mild creamy, slightly sweet, and buttery flavor attributes [63]. However, the flavor of acetoin is considerably weaker than that of butane-2,3-dione. Furthermore, it was proposed that acetoin may reduce the harshness of butane-2,3-dione [76]. Elevated acetoin levels are therefore a further important differentiator between commercial kefir and yogurt. Instead, four new compounds were detected, including acetic acid (8) and three that were unknown (3, 4, and 20). The particular presence of acetic acid in commercial kefir, compared to commercial yogurt, seems to be a major distinguishing feature. Despite some variations, the characteristic spectra of commercial yogurt and kefir are quite similar. As yogurt is only produced by bacteria, and as very similar VOC profiles for commercial yogurt and kefir are seen, it was presumed that commercial kefir is mainly produced by bacteria as well. To investigate this further, quantitative polymerase chain reaction (qPCR) analysis was performed (see Section 2.5).

NNMF-C2 (see Figure 5C), which is characteristic for traditionally made mild kefir, lacks the characteristic peaks for butane-2,3-dione and acetoin, as well as for 2-butanone, associated with milk. The concentration of ethanol had increased, which caused an undesirable spread of the peaks. Ethanol production in kefir was previously attributed to yeast [77], but it was also noted that *Lactococcus* and *Lactobacillus* species possess mild alcohol dehydrogenase activity, which converts acetaldehyde to ethanol. Furthermore, two alcohols were found which were previously reported in kefir [68]: 2-methyl-1-propanol (12) and methyl-1-butanol (18a,b). Since 2-methyl- and 3-methyl-1-butanol have very similar retention times, differentiation is rather difficult, and both compounds were cumulated and are further referred to as ‘methyl-1-butanol’. Moreover, two esters were detected which were previously reported [68]: ethyl acetate (11) and ethyl hexanoate (29). Both esters are composed of ethanol with acetic acid and hexanoic acid, respectively, which have all been identified in this work. NNMF-C2 also reveals the presence of two aldehydes: 3-methylbutanal (13) and hexanal (21). Additionally, five unknown compounds were detected (3, 4, 5, 6, and 26). The retention time and drift times measured for (3) correspond well to pentane, which is not commonly found in kefir. Hydrocarbons, such as pentane, and aldehydes, such as hexanal, are secondary oxidation products which rapidly develop through hydroperoxides, which are primary oxidation products of unsaturated fatty acids [78]. Due to its low odor threshold (5 ppb), hexanal is easily detected and is directly related to oxidative off-flavors [79]. Thus, the presence of hexanal and pentane, especially in traditional mild kefir, may be a consequence of secondary lipid oxidation. It was concluded that the flavor profile is predominantly yeast-related.

NNMF-C1 (see Figure 5D), which is associated with the traditionally made tangy kefir, shows higher concentrations of alcohols, aldehydes, and esters. For all three alcohols (2-methyl-1-propanol (12) and 2-methyl- and 3-methyl-1-butanol (18a,b)), elevated levels (1.6-fold change) are reported compared to the mild kefir. Next to the two esters already found in the mild kefir (ethyl acetate (11) and ethyl hexanoate (29)), a third ester, 3-methylbutyl acetate (24), which consists of 3-methyl-1-butanol and acetic acid, was found. The abundance of ethyl acetate (11) is 3.4-fold higher in C1 than in C2. Three aldehydes were found (2-methyl propanal (7), 3-methylbutanal (13), and 2-methylbutanal (14)), but hexanal was absent. Two additional compounds (23 and 27) were found, which may be another ester and another aldehyde, respectively. The abundance of 3-methylbutanal (13) was elevated 4-fold. To investigate whether the spectra obtained for the traditional kefir varieties were actually yeast-mediated, qPCR was performed.

### 2.5. Identification of Microorganisms Using qPCR

Multiplex TaqMan qPCR was used to analyze three commercial kefir samples as well as six traditional kefir samples (two tangy and four mild samples at 24 and 48 h of fermentation). The development of the multiplex TaqMan qPCR for kefir analysis was previously described by Nejati and coworkers [80]. In total, 11 microorganisms which are commonly found in kefir, including four bacteria (*Lactobacillus (Lb.) kefiranofaciens*, *Lb. kefiri*, *Leuconostoc (Le.) mesenteroides*, and *Lactococcus (Lc.) lactis*), two acetobacters (*Acetobacter (Ac.) orientalis*, *Ac. fabarum*), and five yeasts (*Saccharomyces (S.) cerevisiae*, *Kluyveromayces (Kl.) marxianus*, *Kazachstania (Kz.) turicensis*, *Kz. unispora*, and *Dekkera (D.) anomalous)* were searched for. In the commercial kefir, two bacteria were mainly found, *Le. mesenteroides* and *Lc. lactis*, but yeast and acetobacter were absent. Therefore, the presumption drawn from the VOC profiles, that commercial kefir is mainly produced from bacteria, was confirmed.

For the traditional kefir samples, four bacteria and four yeasts were found using qPCR analysis (see Supplementary Materials Figure S6). Two bacteria (*Le. mesenteroides* and *Lc. lactis*) and one yeast (*Kl. marxianus*) were only found in the tangy samples, and two yeasts were only found in mild kefir (*Kz. unispora* and *D. anomalous*). The presence of isoamyl acetate (24), for example, is likely a fermentation product of *Kl. maxianus* and the presence of hexanal due to *Kz. unispora* or *D. anomalous*. We conclude that the extrapolation of information about microbial consortium based on VOC profile is possible. In further studies, next-generation sequencing should be performed (whole genome sequencing) to further investigate the microbial consortium and to correlate the VOC profiles obtained by optimized HS-GC-IMS.

### 2.6. Further Investigation of the Origin of Hexanal in Kefir Samples

Hexanal is a secondary product of the lipid oxidation of fatty acids. It is an off-flavor (rancidity) which is also detected in sourdough bread [81]. Therefore, further investigation of the hexanal origin in kefir was performed. As shown in the dendrogram based on five NNMF components and five labels (see Supplementary Materials Figure S5), the two mild traditional kefirs can be further separated into two subgroups. When using five NNMF components, the mild kefir variety LS was displayed in NNMF-C2 and the FN variety in NNMF-C4 (data not shown). The comparison of NNMF-C2 and NNMC-C4 shows that hexanal appears in NNMF-C4, which is characteristic for FN (see Supplementary Materials Figure S7A), but it is absent in NNMF-C2 (see Supplementary Materials Figure S7B), which is characteristic for LS. As LS contains *Kz. unispora* but no *D. anomalous*, and FN contains both *Kz. unispora* and *D. anomalous*, it was concluded here that *D. anomalous* was likely causing the hexanal production. To produce a kefir with less off-flavor or rancid taste, a variety excluding *D. anomalous* should therefore be investigated.

A commonly applied method for the identification of relations between the appearance of microorganisms and compounds is the accomplishment of a correlation matrix. In this case, however, the data cannot be sufficiently decorrelated using a correlation matrix. The bacteria *Lb. kefiranofaciens* correlates with *Lb. kefiri* and *Kz. Unispora* (see Supplementary Materials Table S3). Furthermore, *Lb. kefiri* correlates with *Kz. turicensis* and *D. anomalous*. Therefore, when correlating the peak height with the cell number of microorganisms, the results are highly biased. In general, only five substances were found with a correlation of >85%, including ethyl hexanoate and hexanal. Both correlate with *Lb. kefiranofaciens*, *Lb. kefiri*, *Kz. turicensis*, and *D. anomalous*. Using a correlation matrix, the investigation of the hexanal origin was therefore inconclusive. On the contrary, propanol, 2-methyl-1-propanol (isobutanol), and 3-methylbutyl acetate (isoamyl acetate) were correlated solely with *Kl. marxianus*. This comparison demonstrates the power of NNMF for non-targeted VOC profiling of fermented dairy and the evaluation of microbial consortia.

## 3. Materials and Methods

### 3.1. Reagents and Fermented Dairy Samples

Stock solutions of reference compounds were prepared in LC-MS-grade water (HiPerSolv Chromanorm, CAS-No. 7732-18-5 from VWR International GmbH, Germany). Analytical standards, including 2-methyl-1-propanol (CAS-No. 78-83-1), 2-methyl-1-butanol (CAS-No. 137-32-6), 3-methyl-1-butanol (CAS-No. 123-51-3), 3-methylbutanal (CAS-No. 590-86-3), 2-methylbutanal (CAS-No. 96-17-3), 2-methyl propanal (CAS-No. 78-84-2), hexanal (CAS-No. 66-25-1), 2-butanone (CAS-No. 78-93-3), 3-hydroxybutan-2-one (CAS-No. 513-86-0), 2-pentanone (CAS-No. 107-87-9), 2-heptanone (CAS-No. 110-43-0), 2-nonanone (CAS-No. 821-55-6), and butane-2,3-dione (CAS-No. 431-03-8), as well as 3-methylbutyl acetate (CAS-No. 123-92-2) and ethyl acetate (CAS-No. 141-78-6), were purchased from Sigma-Aldrich Chemie GmbH, Taufkirchen, Germany). Ethanol (CAS-No. 64-17-5), 2-propanol (CAS-No. 71-23-8), acetone (CAS-No. 67-64-1), and acetic acid (CAS-No. 64-19-7) were purchased from VWR International GmbH, Darmstadt, Germany, hexanoic acid (CAS-No. 142-62-1) from Alfa Aesar, Heysham, UK, butyric acid (CAS-No. 107-92-6) from Merck KGaA, Darmstadt, Germany, and 2,3-pentandione (CAS-No. 600-14-6) and ethyl hexanoate (CAS-No. 123-66-0) from Acros Organics by Thermo Fisher GmbH, Kandel, Germany. All analytical standards were purchased at ≥98% purity.

In total, 76 fermented dairy samples were collected for this work, including 16 commercial kefir and 18 commercial yogurt samples purchased in Germany, as well as 42 traditionally produced (grain-based) kefir samples provided by TU Berlin, Germany. Additional information about the sample composition and origin are provided in Appendix A, Table A1, Table A2 and Table A3. Five traditional kefirs (PN1, PN2, PN3, FN, and LS) used in this study were from Berlin, Germany, and had been propagated by three different households. Kefirs FN and LS were originated from PN kefir and were separately propagated for at least three years. Kefirs PN1, PN2, and PN3 were propagated by one household in different batches for at least three years. For sub-culturing in the current study, grains (100 g L^−1^) were inoculated into UHT-sterilized cow milk (1.5% fat, Alnatura, Darmstadt, Germany) followed by incubation at 25 °C for about 48 h. Kefir preparation was performed three times, each time two months apart. Per kefir grain, up to four independent fermentations were completed, and samples were taken between 23 h and 48 h of incubation. Samples were harvested after 1 day, 1.5 days, and 2 days, resulting in 20 mild (FN, LS) and 22 tangy (PN) varieties. In this manuscript, samples which were harvested between 20 and 30 h of fermentation are referred to as ‘early harvest’, and samples that were harvested between 30 and 50 h as ‘late harvest’. Commercial samples were stored according to producer’s specifications and analyzed prior to expiry date. Traditionally produced samples were stored and shipped at −18 °C in 50 mL falcon tubes. For the isolation of VOCs, 0.5 g of kefir or yogurt was transferred into a 20 mL headspace vial, mixed with 0.5 mL saturated sodium chloride solution, and sealed with a 2 mm silicon cap. Subsequently, the samples were incubated for 20 min at 75 °C and 750 rpm. Following incubation, 1 mL of headspace volume was automatically injected into the heated injector of the GC-IMS using a heated syringe. Two GC-IMS systems were used for analysis: a reference HS-GC-IMS/MS dual detection system based on a standard OEM-IMS cell and a HS-GC-HTIMS prototype with adjustable drift tube temperature. The determination of the headspace volatiles was performed in duplicate.

### 3.2. Analysis of Microbial Composition of Kefirs

DNeasy PowerSoil Pro (Qiagen, Germany) was used to extract microbial DNA from all kefirs in both grain and beverage fractions. One mL each of commercial kefirs or grain-free traditional kefirs was applied to extract the DNA in the kefir beverage fraction. The extracted DNAs were subjected to quantification of microbial community by qPCR by applying the developed method in our previous work [80]. In total, eleven microorganisms which are commonly found in kefir were tested for, including four bacteria (*Lb. kefiranofaciens*, *Lb. kefiri*, *Le. mesenteroides*, *Lc. lactis*), two acetobacters (*Ac. orientalis*, *Ac. fabarum*), and five yeasts (*S. cerevisiae*, *Kl. marxianus*, *Kz. turicensis*, *Kz. unispora*, and *D. anomalous*).

### 3.3. Metabolite Analysis (Acetic Acid Quantification)

The content of several carbohydrates and common degradation products, including acetic acid, of the beverages of commercial and traditional kefirs was analyzed with the Cedex Bio HT Analyzer (Roche Diagnostics International AG, Risch-Rotkreuz, Switzerland). Before analysis, the samples were centrifuged at 12,000 rpm for 5 min (4 °C), and the supernatants were applied to metabolite analysis.

### 3.4. HS-GC-MS System

GC-MS measurements were performed on a Shimadzu single quadrupole GCMS-QP2020 NX gas chromatograph–mass spectrometer (Shimadzu, Kyoto, Japan). Static headspace injection was carried out with a CombiPAL autosampler (CTC Analytics AG, Zwingen, Switzerland). After sample incubation at 75 °C for 20 min, 1 mL headspace volume of sample was injected with a gastight 2.5 mL heatable syringe (Hamilton, Reno, NV, USA) into the GC injector operated at 200 °C in split mode with a split ratio of 1:10. The syringe was heated constantly at 80 °C and flushed for 3 min before each injection cycle to avoid condensation effects and carry-over. The GC was equipped with a low-polarity capillary column (DB-XLB, 30 m × 0.25 mm × 0.25 μm, Agilent Technologies, Santa Clara, CA, USA) and operated with a column gas flow of 0.95 mL min^−1^ of helium. At 40 °C initial temperature, the oven program started, followed by a temperature ramp of 10 °C min^−1^ to 140 °C, at which point it was increased by 20 °C min^−1^ to 200 °C, resulting in a total run time of 13 min. The analytical column’s output was fed into an MS, equipped with an electron impact (EI) ion source operated at 230 °C.

### 3.5. Reference HS-GC-MS/IMS System Based on Standard OEM-IMS Cell

Reference measurements were performed on an HS-GC-MS/IMS dual-detection system consisting of an Agilent 6890 GC (Agilent Technologies Deutschland GmbH, Waldbronn, Germany) coupled to an Agilent 5973 mass spectrometer (EI mode) (Agilent Technologies Deutschland GmbH) and an OEM-IMS cell (Gesellschaft für Analytische Sensorsysteme mbH, Dortmund, Germany). In our previous work, this system was successfully applied for the quantification of allergenic fragrance compounds in complex cosmetic applications [23], as well as for the volatilomic profiling of citrus juices [64].

Static headspace injection was carried out with a CombiPAL autosampler (CTC Analytics AG, Zwingen, Switzerland). After sample incubation, 1 mL headspace volume of sample was injected with a gastight 2.5 mL heatable syringe (Hamilton, Reno, NV, USA) into the GC injector operated at 250 °C in split mode with a split ratio of 1:10. The syringe was heated constantly at 80 °C and flushed for 3 min before each injection cycle to avoid condensation effects and carry-over. The GC was equipped with a (5%-phenyl)-methylpolysiloxane capillary column (ZB-5 ms, 30 m × 0.25 mm × 0.25 μm, Phenomenex Inc., Torrance, CA, USA) and operated with a helium carrier gas flow of 4 mL min^−1^. At 40 °C initial temperature, the oven program started followed by a temperature ramp of 10 °C min^−1^ to 140 °C, resulting in a total run time of 10 min for the HS-GC-IMS/MS system trials. At the end of the analytical column, the gas flow was split at the splitter plate by two identical retention gaps (1 m length and 0.1 mm inner diameter) and led to the two coupled detectors via heated transfer lines operated at 200 °C (IMS) and 250 °C (MS), respectively. The IMS consisted of a ^3^H-radioactive ionization source and a drift tube (9.8 cm length) operated at 90 °C and 5 kV drift voltage under a constant nitrogen drift gas flow of 150 mL min^−1^ of 99.9999% gas purity. Spectra were recorded in positive polarity mode with a repetition rate of 21 ms, an injection pulse width of 150 μs, and a blocking and injection voltage of 70 mV and 2500 mV, respectively. To reduce the data size, six single spectra were averaged. The MS was equipped with an EI ion source operated at 230 °C with 70 eV ionization energy and a single quadrupole at 150 °C.

### 3.6. GC-HTIMS Prototype with Adjustable Drift Tube Temperature

All GC-IMS analyses presented here were performed on a HTIMS prototype manufactured by Gesellschaft für Analytische Sensorsysteme mbH (Dortmund, Germany) coupled with an Agilent 6890N gas chromatograph (Agilent Technologies, Santa Clara, CA, USA).

The system was equipped with a CombiPal GC autosampler (CTC Analytics AG, Zwingen, Switzerland) with a headspace sampling unit and a 2.5 mL gas-tight heatable syringe. For the analysis, a headspace volume of 1 mL was sampled at a speed of 350 μL s^−1^ and a syringe temperature of 80 °C to avoid condensation effects. Before each analysis, the syringe was automatically flushed with a stream of nitrogen for 5 min to avoid cross contamination. Injection was performed into a split/splitless injector, operated at 150 °C in split mode (split 1:10). Chromatographic separation was performed on an HP-5 capillary column (material: (5% phenyl)-methylpolysiloxane; operating temperature: −60 °C to 325 °C/350 °C; SN: USB345942H) with a 30 m × 0.32 mm × 0.25 μm film thickness (Agilent Technologies, Santa Clara, CA, USA). Nitrogen of 99.99% purity was used as the carrier gas at an initial column flow of 1.5 mL min^−1^. The pressure was set to a constant 6.7 psi. The GC oven temperature was programmed as follows: the GC oven was preheated to 40 °C and increased to 140 °C at 10 °C min^−1^, which correlates to 10 min of GC runtime.

Following gas-chromatographic separation, the analytes were ionized in the IMS ionization chamber by a ^3^H ionization source (300 MBq activity). The drift tube length was 5.3 cm and was operated at a constant voltage of 2.5 kV with a nitrogen flow of 150 mL min^−1^. The gas flow was controlled by a mass flow controller (Voegtlin Instruments AG, Aesch, Switzerland). The prototype allows for temperature variation of the drift tube between 60 °C and 180 °C. The IMS cell was operated in positive ion mode. Each spectrum was the average of six scans obtained by using injection pulse widths of 100 μs, sampling frequencies of 228 kHz, and repetition rates of 21 ms. The data were collected using LAV software version 2.2.1 from Gesellschaft für Analytische Sensorsysteme mbH (Dortmund, Germany).

### 3.7. Ambient Pressure Measurements

The ambient pressure was measured using a portable data logger DL-220THP (Conrad Electronics SE, Hirschau, Germany). The product specific air pressure measuring range is 300 to 1200 hPa with an accuracy of 3 hPa.

### 3.8. Data Preprocessing

Prior to the multivariate data analysis, data preprocessing was performed by MATLAB 2020a software (The MathWorks Inc., Natick, MA, USA) and the Bioinformatics MATLAB toolbox. Firstly, all IMS spectra were exported from the LAV Software (each spectrum as a two-dimensional matrix of retention time (R) and drift time (D) with signal intensities). These individual matrices were concatenated into a three-dimensional (3D) array (samples (S) × R × D). For data reduction, the mean was calculated from five consecutive spectra. Furthermore, drift times were normalized to the RIP position. Following the alignment, spline interpolation was applied to create a uniform RIP-relative drift time axis. Regions of relevant signal were chosen, and spectra were cropped accordingly. The new drift time axis was set from 1.03 to 2 RIP relative, and the new retention time axis from 150 s to 600 s. To improve the signal-to-noise ratios of all spectra, a baseline correction was applied to drift time and retention time direction. Subsequently, the duplicate measurements were averaged for each sample. As most algorithms, such as PCA and also NNMF, require 2D matrices, the three-dimensional matrix (S × R × D) was unfolded row-wise into a two-dimensional matrix (S × RD) (see Section 3.9 for further processing).

### 3.9. Chemometric Data Analysis and Software

Multivariate data analysis was performed with an author’s own MATLAB routines [36] and the Statistics and Machine Learning MATLAB toolbox with MATLAB 2019a (The MathWorks Inc., Natick, MA, USA). Spectra obtained from fermented dairy samples (total 76) were divided into a training set (61 samples), consisting of 15 commercial yogurt, 13 commercial kefir, 16 mild kefir, and 17 tangy kefir samples, and a test set, consisting of 3 commercial yogurt, 3 commercial kefir, 4 mild kefir, and 5 tangy kefir samples (15 samples), resulting in a train–test split of 80% to 20%. PCA and NNMF were applied as unsupervised exploratory techniques for dimension reduction and visualization of potential class differences of HS-GC-IMS data. To visualize group similarities calculated via PCA and NNMF analysis, HCA was performed using the Euclidean distance and the Ward method. Furthermore, score and loading plots of the NNMF analysis of IMS data were used to identify VOCs responsible for class separation. For semi-quantitative evaluation of each substance, detected via NNMF analysis, the signals above the plateau of the local minimum were integrated using a self-written MATLAB routine. The peak volume was subsequently normalized to the pre-selected GC-IMS peak area. To visualize the similarities between the peak volume (arbitrary units) of kefir and yogurt samples, a heatmap was generated.

Compared to unsupervised techniques, which provide predictions without labels or target variables, supervised techniques aim to build models able to predict target variables. In supervised learning, several data points or samples are described using predictor variables, or features, and target variables. For classification tasks, the scores obtained by the unsupervised exploratory analysis are combined with subsequent supervised pattern recognition techniques to distinguish samples according to defined categories. Here, LDA, *k*NN, and SVM were applied as supervised classification models based on the PCA or NNMF scores obtained from HS-GC-IMS measurements. Hereby, PCA-LDA maximizes the interclass variance and *k*NN assigns the category most common among the *k*-nearest neighbors, hence representing a very simple and only distance-based method for discriminant classification [82]. The class membership of an object is determined by the class membership of the *k*-nearest neighbors, usually identified by the Euclidean distance [83]. For *k*NN, five nearest neighbors were selected as a classification criterion. To avoid overfitting of the applied models, the choice of a reasonable number of PCs is crucial. The optimum number was determined by increasing the number of PCs stepwise until the prediction error obtained by CV increased.

The disadvantage of using PCA-based methods is that the correlation between dependent and independent variables is not considered during PCA analysis, which can result in a loss of the information included in higher PCs [37]. An alternative is provided by applying PLS, where the scores are calculated by considering the relationship between the independent and dependent variables. In this context, PLS-DA is a special case of classification, as it basically uses a regression approach with class boundaries instead of single values, as in quantitative regressions.

## 4. Conclusions

In this work, an IMS protocol for the analysis of fermented dairy samples was developed. By increasing the drift tube temperature in a prototypic HTIMS, an improvement in the peak separation of acetic acid and diacetyl, two key compounds in fermented dairy, was achieved. Furthermore, it was shown that PCA and NNMF analysis are powerful pattern recognition and data reduction techniques for the discrimination and classification of fermented dairy samples. Through backwards projection of NNMF loadings, 22 VOCs were identified, and conclusions were able to be drawn about the bacterial composition. The use of qPCR analysis showed that the extrapolation of information about microbial consortia based on VOC profiles in combination with machine learning is possible. This optimized HS-GC-IMS protocol is suitable for real-time measurements and could therefore be used for systematic and effective quality control of fermented milk products to ensure product safety and quality. By monitoring the parameters of all the feed and in-process components as well as the production process, contaminants may be detected early, and the total safety of every batch can be guaranteed.

## Figures and Tables

**Table 1:** Prediction results for 4 labels. (L)DA = (linear) discriminant analysis; *k*NN, *k*-nearest neighbor classification, *k* = number of neighbors; SVM = support vector machine.

Pattern Recognition and Data Reduction Technique	Supervised Method	CV Error Rate (%)	Prediction Accuracy (Posterior) (%)
PCA (PC 1 to 4)	LDA	0	100
	*k*NN (*k* = 5)	0	100
	SVM	3.3 (2 out of 61)	100
PLS (PLS 1 to 4)	DA	1.6 (1 out of 61)	100
PLS (PLS 1 to 5)	DA	0	100
NNMF (C 1 to 4)	LDA	3.3 (2 out of 61)	87 (13 out of 15)
	*k*NN (*k* = 5)	0	100
	SVM	5.0 (3 out of 61)	100

**Table 2:** Prediction results for 5 labels. (L)DA = (linear) discriminant analysis; *k*NN, *k*-nearest neighbor classification, *k* = number of neighbors; SVM = support vector machine.

Pattern Recognition and Data Reduction Technique	Supervised Method	CV Error Rate (8-Fold CV) (%)	Prediction Accuracy (Posterior) (%)
PCA (PC 1 to 4)	LDA	1.6 (1 out of 61)	0
	*k*NN (*k* = 5)	0	0
	SVM	6.6 (4 out of 61)	0
PCA (PC 1 to 5)	LDA	0	100
	*k*NN (*k* = 5)	0	100
	SVM	9.8 (6 out of 61)	93 (14 out of 15)
PLS (PLS 1 to 4)	DA	0	100
PLS (PLS 1 to 5)	DA	0	100
NNMF (C 1 to 5)	LDA	5.0 (3 out of 61)	93 (14 out of 15)
	*k*NN (*k* = 5)	0	100
	SVM	8.2 (5 out of 61)	87 (13 out of 15)

**Table 3:** Volatile organic compounds (VOCs) detected in kefir by gas chromatography–ion mobility spectroscopy (GC-IMS) and GC–mass spectrometry (GC-MS). RS = reference substance.

#	Compound	Retention Time Start (s)	Molar Mass (g mol^−1^)	Odor Descriptor	Identification Method
1	Ethanol	150.1	46	Dry, dust [68]	RS
2	Acetone	155.6	58	Earthy, fruity, wood pulp, hay [68]	RS, MS
3	Tentatively pentane	155.6	72	Faint gasoline-like [69]	RS
4	Unknown	155.9			
5	Tentatively propanol	167.6	60	Mild, alcohol-like [70]	RS
6	Unknown	167.6			
7	2-methyl propanal (isobutyraldehyde)	167.6	58	Faint gasoline-like, natural gas [71]	RS, MS
8	Acetic acid	169.9	88	Vinegar, peppers, green, fruity, floral, sour [68]	RS, MS
9	Butane-2,3-dione (diacetyl)	174.0	86	Buttery, strong [68]	RS, MS
10	2-butanone	178.0	80	Buttery, sour milk, etheric [68]	RS
11	Ethyl acetate	182.2	60	Solvent, pineapple, fruity, apples [68]	RS, MS
12	2-methyl-1-propanol (isobutanol)	188.1	74	Malty [68]	RS, MS
13	3-methylbutanal	198.7	86	Malty, cheesy, green, dark chocolate, cocoa [68]	RS, MS
14	2-methylbutanal	203.3	86	Malty, dark chocolate, almond, cocoa, coffee [68]	RS
15	2,3-pentandione	216.3	100	Creamy, cheesy, oily, sweet buttery, caramellic [68]	RS
16	2-pentanone	214.2	86	Orange peel, sweet, fruity [68]	RS
17	3-hydroxybutan-2-one (acetoin)	226.7	88	Bland, yogurt-like [72]	RS
18	2-methyl-1-butanol	244.4	88	Penetrating, alcohol, wine-like, plastic [68]	RS
19	3-methyl-1-butanol (isoamyl alcohol)	239.4	88	Fresh cheese, breathtaking, alcoholic, fruity, grainy, solvent-like, floral, malty [68]	RS, MS
20	Butyric acid	264.3	88	Unpleasant, similar to vomit or body odor [73]	RS
21	Unknown	282.1			
22	Hexanal	286.3	100	Green, slightly fruity, lemon, herbal, grassy, tallow [68]	RS
23	Unknown	299.8			
24	Unknown	320.5			
25	3-methylbutyl acetate (isoamyl acetate)	355.1	130	Fruity, banana, candy, sweet, apple peel [68]	RS
26	2-heptanone	369.2	114	Blue cheese, spicy, Roquefort [68]	RS
27	Unknown	378.7			
28	Unknown	427.3			
29	Hexanoic acid (caproic acid)	443.7	116	Sweaty, cheesy, sharp, goaty, bad breath, acidic [68]	RS
30	Ethyl hexanoate	474.0	144	Fruity, malty, young cheese, moldy, apple, green, orange, pineapple, banana [68]	RS
31	2-nonanone	570.8	142	Malty, fruity, hot milk, smoked cheese, lipid metabolism [68]	RS

## Data Availability

Data are available in a publicly accessible repository that does not issue DOIs. Publicly available datasets were analyzed in this study. These data can be found here: https://drive.google.com/drive/folders/1jEdutft-XwO4rj4xzEL5wX6ubDBgTaIn?usp=sharing (accessed on 1 February 2022).

## Supplementary Materials

The following supporting information can be downloaded at: https://www.mdpi.com/article/10.3390/metabo12040299/s1. Table S1: IMS drift time (center) and GC retention time (start) obtained for ethanol, acetic acid, ethyl acetate, and diacetyl using reference substances. Between one (for diacetyl) and four (for acetic acid) characteristic peaks were obtained per substance. Table S2: IMS drift time (center) and GC retention time (start) for characteristic peaks found in optimized HS-GC-IMS analysis at an IMS cell temperature of 120 °C. Table S3: Correlation matrix for qPCR results. Figure S1: Mass spectra of (A) acetic acid and (B) ethyl acetate. Spectra were provided by SDBSWeb [84]. Acetic acid has its main peaks at *m*/*z* ratios of 43 (100.0% relative intensity), 45 (90.4% rel. int.), and 60 (74.8% rel. int.). Furthermore, some peaks with a relative intensity below 20% can be found. Ethyl acetate has its main peak at a *m*/*z* ratio of 43 (100%). Furthermore, three peaks with relative intensities between 13.7% and 14.9% at *m*/*z* ratios of 29, 45, and 61 are found. Further peaks show relative intensity below 10%. Figure S2: IMS spectra of (A) ethanol, (B) acetic acid, (C) ethyl acetate, (D) diacetyl, (E) commercial Mueller kefir, and (F) traditionally fermented kefir LS at 48 h. For comparison, the three peaks of acetic acid (aa_1, aa_2, and aa_3) are marked with boxes. Auto sampler incubation time: 20 min, incubation temperature: 75 °C. Figure S3: Three-dimensional scatter plot of processed HS-GC-IMS spectra of fermented dairy determined by PCA. Shown are PC1, PC2, and PC4, which explain 85.2% of the total variance. PCA was obtained using the full spectrum of HS-GC-ISM data. Figure S4: Heatmap of semi-quantitative results of NNMF analysis. The signals above the plateau of the local minimum were integrated using a self-written MATLAB routine. The values represent the peak volume in arbitrary units. NNMF-C1 shows characteristics patterns of traditional tangy kefir, NNMF-C2 of traditional mild kefir, NNMF-C3 of commercial yogurt, and NNMF-C4 of commercial kefir. Figure S5: Dendrogram based on NNMF analysis (C1–C5). A detailed description of the sample data is provided in Appendix A, Table A1, Table A2 and Table A3. Figure S6: qPCR results for traditional kefir samples. Figure S7: NNMF-C2 (top) and NNMF-C4 (bottom) for NNMF analysis using 5 components. The white error shows the presence of hexanal (dimer peak).

## Appendix A

Appendix A provides additional information about the sample composition and origin of commercial kefir samples, traditional kefir samples, and commercial yogurt samples.

**Table A1:** Commercial kefir (ck) samples were purchased in local food stores in Germany. The manufacturer, fat content, and organic certification, as well as the supplementation with *L. acidophilus* (A) and *B. bifidus* (B), are listed.

Sample Name	Manufacturer	Fat Content	Organic	Supplementary Cultures
ck01	Alnatura	1.50%	x	
ck03	Andechser expired	1.50%	x	
ck02	Andechser (M3) frozen	1.50%	x	
ck04	Berchtesgadener	1.50%	x	
ck05	Berchtesgadener	1.50%		
ck06	Brandenburg (M1) frozen	3.50%		
ck07	Gut & Günstig	1.50%		
ck08	GutBio	1.50%	x	
ck09	Müller (M2) frozen	1.50%		
ck10	Müller	1.50%		
ck11	Quarki	2.00%		
ck12	Schrozberger	1.50%	x	AB
ck13	Starter_culture (23h)	3.50%	x	
ck15	Starter_culture (48h)	3.50%	x	
ck14	Starter_culture (23h) frozen	3.50%	x	
ck16	Starter_culture (48h) frozen	3.50%	x	

**Table A2:** Traditional kefir (tk) samples were provided by TU Berlin. Five traditional kefirs (PN1, PN2, PN3, FN, and LS) used in this study were from Berlin, Germany, which have been propagated by three different households. Kefirs FN and LS were originated from PN kefir and were separately propagated for at least three years. Kefirs PN1, PN2, and PN3 were propagated by one household in different batches for at least three years.

#	Kefir Grain	Harvest Time (h)	Run	Data Obtained in
FN_R2_23h	FN	23	R2	02/2021
FN_R1_24h	FN	24	R1	12/2020
FN_R2_46h	FN	46	R2	02/2021
FN_R1_48h	FN	48	R1	12/2020
FN_R3_24h	FN	24	R3	04/2021
FN_R3_37h	FN	37	R3	04/2021
FN_R3_48h	FN	48	R3	04/2021
FN_R4_24h	FN	24	R4	04/2021
FN_R4_37h	FN	37	R4	04/2021
FN_R4_48h	FN	48	R4	04/2021
LS_R2_23h	LS	23	R2	02/2021
LS_R1_24h	LS	24	R1	12/2020
LS_R2_46h	LS	46	R2	02/2021
LS_R1_48h	LS	48	R1	12/2020
LS_R3_24h	LS	24	R3	04/2021
LS_R3_37h	LS	37	R3	04/2021
LS_R3_48h	LS	48	R3	04/2021
LS_R4_24h	LS	24	R4	04/2021
LS_R4_37h	LS	37	R4	04/2021
LS_R4_48h	LS	48	R4	04/2021
PN1_R2_23h	PN1	23	R2	02/2021
PN1_R2_46h	PN1	46	R2	02/2021
PN1_R3_24h	PN1	24	R3	04/2021
PN1_R3_37h	PN1	37	R3	04/2021
PN1_R3_48h	PN1	48	R3	04/2021
PN1_R4_24h	PN1	24	R4	04/2021
PN1_R4_37h	PN1	37	R4	04/2021
PN1_R4_48h	PN1	48	R4	04/2021
PN2_R2_23h	PN2	23	R2	02/2021
PN2_R2_46h	PN2	46	R2	02/2021
PN2_R3_24h	PN2	24	R3	04/2021
PN2_R3_37h	PN2	37	R3	04/2021
PN2_R4_24h	PN2	24	R4	04/2021
PN2_R4_37h	PN2	37	R4	04/2021
PN3_R2_23h	PN3	23	R2	02/2021
PN3_R2_46h	PN3	46	R2	02/2021
PN3_R3_24h	PN3	24	R3	04/2021
PN3_R3_37h	PN3	37	R3	04/2021
PN3_R3_48h	PN3	48	R3	04/2021
PN3_R4_24h	PN3	24	R4	04/2021
PN3_R4_37h	PN3	37	R4	04/2021
PN3_R4_48h	PN3	48	R4	04/2021

**Table A3:** Commercial yogurt (cy) samples were purchased in local food stores in Germany. The manufacturer, fat content, and organic certification, as well as the supplementation with *L. acidophilus* (A), *B. bifidus* (B), and *L. casei* (C), are listed.

Sample Name	Manufacturer	Fat Content	Organic	Supplementary Cultures
cy1	Andechser	0.10%	x	AB
cy2	Andechser	3.80%	x	AB
cy3	Berchtesgadener	3.50%	x	AB
cy4	Berchtesgadener	3.90%		AB
cy5	Gut & Günstig	1.80%		C
cy6	GutBio	3.80%	x	
cy7	Ja!	1.50%		
cy8	Milsani	1.80%		C
cy9	Schrozberger	1.80%	x	AB
cy10	Schrozberger	3.50%	x	AB
cy11	Schrozberger_ABC	3.50%	x	ABC
cy12	Schwalbenhof	3.50%	x	
cy15	Söbbeke_1	1.50%	x	
cy16	Söbbeke_2	1.50%	x	
cy18	Söbbeke_ABC	3.80%	x	ABC
cy17	Söbbeke	3.80%	x	
cy13	Starter_culture (13h)	3.50%	x	
cy14	Starter_culture (13h) frozen	3.50%	x	
