# Supplementary material for: Volatilomics-Based Microbiome Evaluation of Fermented Dairy by Prototypic Headspace-Gas Chromatography–High-Temperature Ion Mobility Spectrometry (HS-GC-HTIMS) and Non-Negative Matrix Factorization (NNMF)
Source: Metabolites. 2022 Mar 28;12(4):299. doi: 10.3390/metabo12040299 (PMC9025153; doi:10.3390/metabo12040299)
Supplement: Supplementary file 1 [file metabolites-12-00299-s001.zip › metabolites-1619353-supplementary.pdf]

metabolites

MDPI

Table S1. IMS drift time (center) and GC retention time (start) obtained for ethanol, acetic acid, ethyl acetate, and diacetyl using reference substances. Between one (for diacetyl) and four (for acetic acid) characteristic peaks were obtained per substance.

Compound, Peak Number	IMS Drift Time Center [RI-Prel]	GC Retention Start Time [s]
ethanol, peak 1	1.052	90.0
ethanol, peak 2	1.141	90.4
acetic acid, peak 1	1.114	116.9
acetic acid, peak 2	1.393	115.8
acetic acid, peak 3	1.165	107.8
acetic acid, peak 4	1.049	107.5
ethyl acetate, peak 1	1.115	110.8
ethyl acetate, peak 2	1.394	110.3
diacetyl	1.164	109.1

Table S2. IMS drift time (center) and GC retention time (start) for characteristic peaks found in optimized HS-GC-IMS analysis at an IMS cell temperature of 120 °C.

Compound, Peak Number	IMS Drift Time Center [RI-Prel]	GC Retention Start Time [s]
ethanol, peak 1	1.154	150.3
acetic acid, peak 1	1.051	169.3
acetic acid, peak 2	1.178	170.6
ethyl acetate, peak 1	1.110	183.1
ethyl acetate, peak 2	1.430	181.6
diacetyl, peak 1	1.111	174.0
heterodimer	1.231	173.7
2-butanone, peak 1	1.057	178.1
2-butanone, peak 2	1.322	178.1
acetoin	1.176	155.3

Table S3. Correlation matrix for qPCR results.

	A	B	C	D	E	F	G	H
Lb. kefiranofaciens (A)	1							
Lb. kefiri (B)	0,811	1						
Le. mesenteroides (C)	-0,403	-0,300	1					
Lc. lactis (D)	-0,547	-0,350	0,505	1				
Kl. marxianus (E)	0,004	-0,089	0,214	-0,293	1			
Kz. turicensis (F)	0,751	0,821	-0,328	-0,328	-0,191	1		
Kz. unispora (G)	0,855	0,579	-0,530	-0,502	-0,360	0,634	1	
D. anomalous (H)	0,794	0,929	-0,337	-0,320	-0,229	0,970	0,651	1

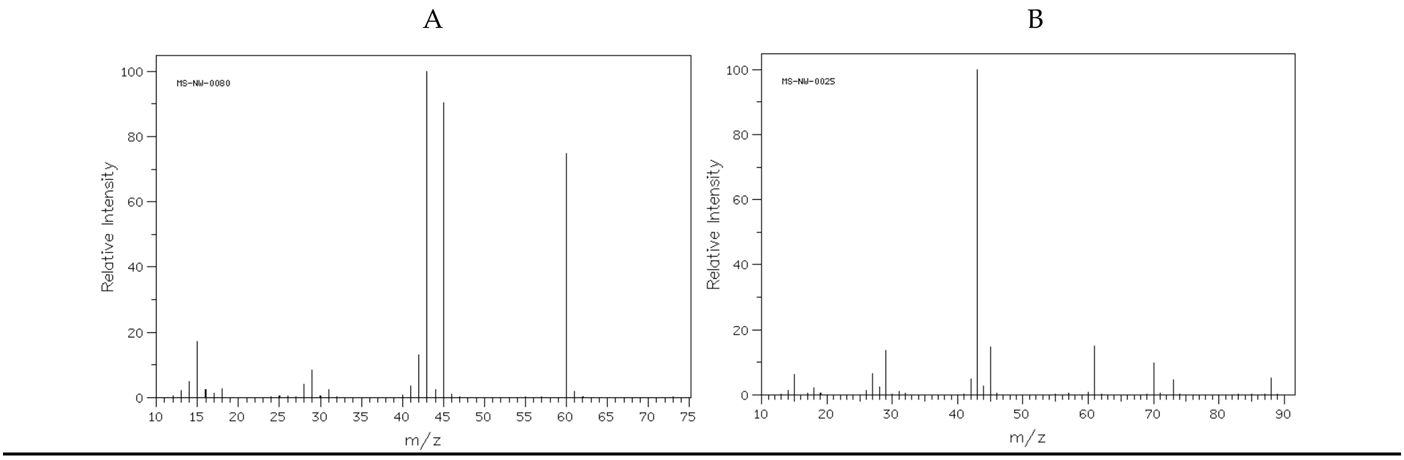

Figure S1. Mass spectra of (**A**) acetic acid and (**B**) ethyl acetate. Spectra were provided by SDBSWeb [78]. Acetic acid has its main peaks at a m/z ratio of 43 (100.0% relative intensity), 45 (90.4% rel. int.) and 60 (74.8% rel. int.). Furthermore, some peaks with a relative intensity below 20% can be found. Ethyl acetate has its main peak at a m/z ratio of 43 (100%). Furthermore, three peaks with relative intensities between 13.7% and 14.9% at m/z ratios of 29, 45 and 61 are found. Further peaks show relative intensity below 10%.

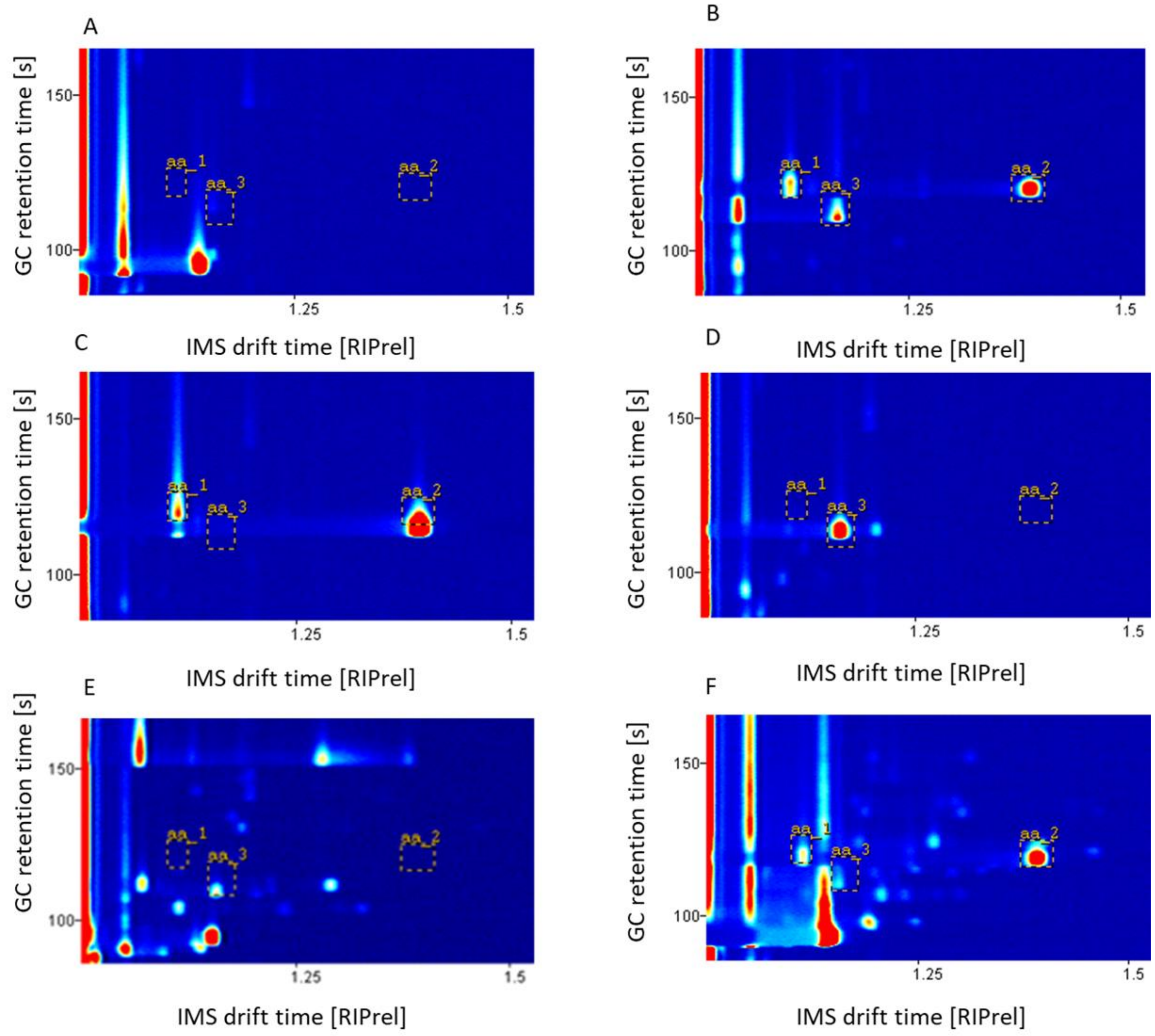

Figure S2. IMS spectra of (**A**) ethanol, (**B**) acetic acid, (**C**) ethyl acetate, (**D**) diacetyl, (**E**) commercial Mueller-kefir and (**F**) traditionally fermented kefir LS at 48 hours. For comparison, the three peaks of acetic acid (aa_1, aa_2 and aa_3) are marked with boxes. Auto sampler incubation time: 20 min, incubation temperature: 75 °C.

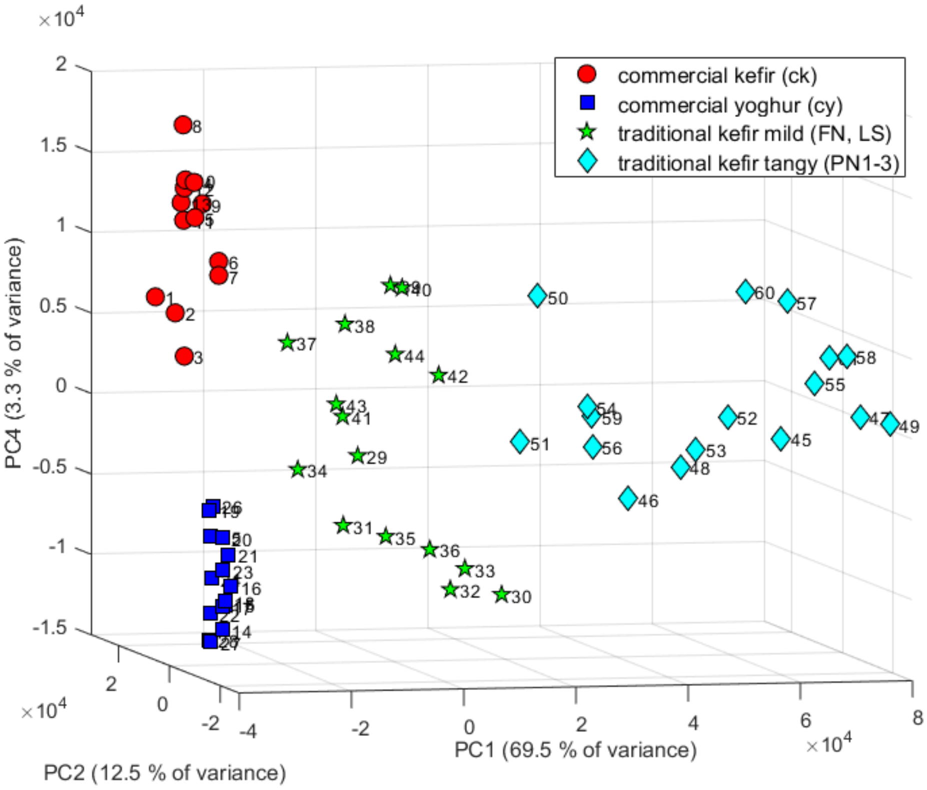

Figure S3. 3D scatter plot of processed HS-GC-IMS spectra of fermented dairy determined by PCA. Shown are PC1, PC2 and PC4, which explain 85.2% of the total variance. PCA was obtained using the full spectrum of HS-GC-ISM data.

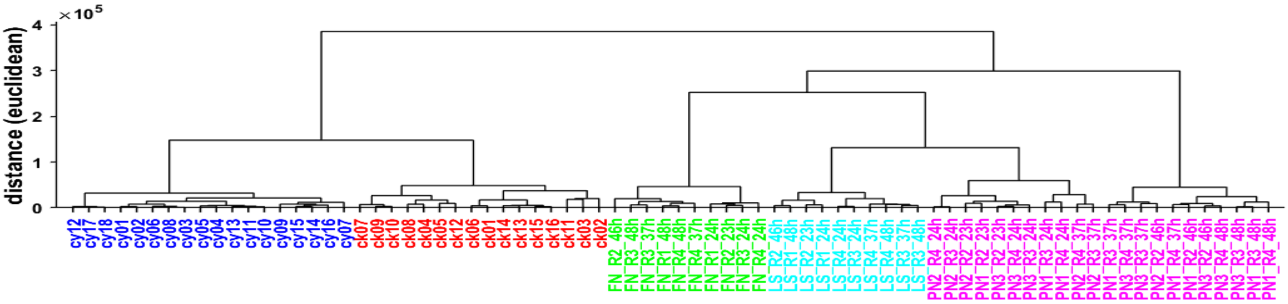

Figure S4. Dendrogram based on NNMF analysis (C1-C5). A detailed description of the sample data is provided in the appendix Table A1 to A3.

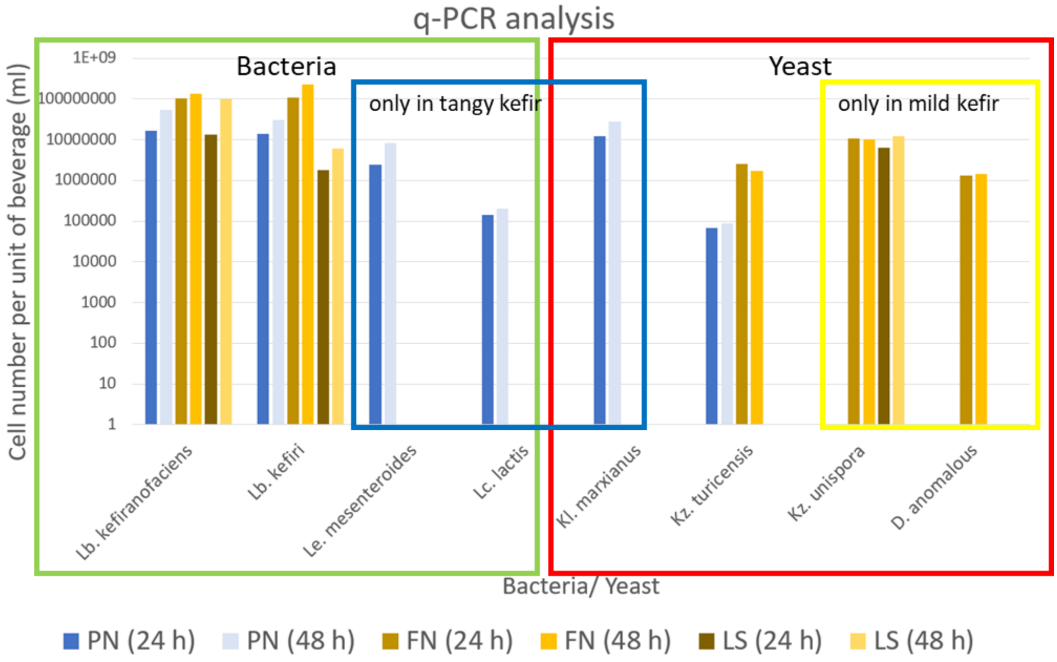

Figure S5. qPCR results for traditional kefir samples: Tangy kefir samples are displayed in blue and mild kefir samples (LS and FN) are shown in yellow. Bacteria are marked with a green box and yeast with a red box.

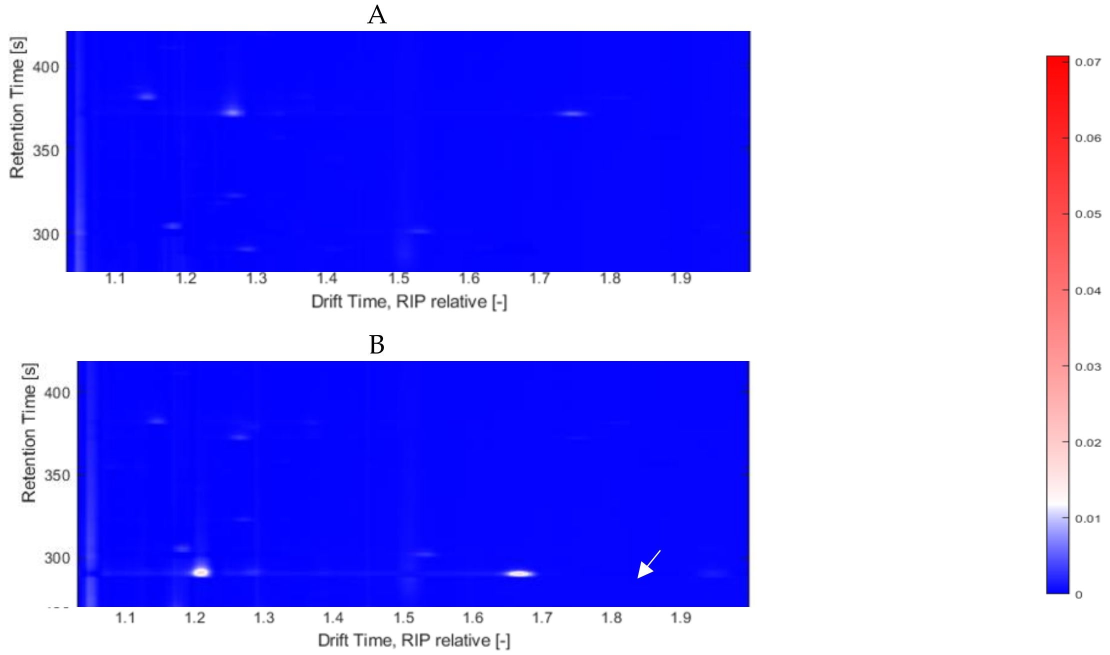

Figure S6. NNMF-C2 (top) and NNMF-C4 (bottom) for NNMF analysis using 5 components. The white error shows the presence of hexanal (dimer peak).
